# Functional Characterization of the Cell Division Gene Cluster of the Wall-less Bacterium *Mycoplasma genitalium*

**DOI:** 10.3389/fmicb.2021.695572

**Published:** 2021-09-13

**Authors:** Carlos Martínez-Torró, Sergi Torres-Puig, Marina Marcos-Silva, Marta Huguet-Ramón, Carmen Muñoz-Navarro, Maria Lluch-Senar, Luis Serrano, Enrique Querol, Jaume Piñol, Oscar Q. Pich

**Affiliations:** ^1^Departament de Bioquímica i Biologia Molecular, Institut de Biotecnologia i Biomedicina, Universitat Autònoma de Barcelona, Barcelona, Spain; ^2^EMBL/CRG Systems Biology Research Unit, Centre for Genomic Regulation (CRG), The Barcelona Institute of Science and Technology, Barcelona, Spain; ^3^Laboratori de Recerca en Microbiologia i Malalties Infeccioses, Institut d’Investigació i Innovació Parc Taulí (I3PT), Hospital Universitari Parc Taulí, Universitat Autònoma de Barcelona, Sabadell, Spain

**Keywords:** mycoplasmas, cell division, regulation, single cell analysis, FtsZ localization, cell cycle

## Abstract

It is well-established that FtsZ drives peptidoglycan synthesis at the division site in walled bacteria. However, the function and conservation of FtsZ in wall-less prokaryotes such as mycoplasmas are less clear. In the genome-reduced bacterium *Mycoplasma genitalium*, the cell division gene cluster is limited to four genes: *mraZ*, *mraW*, MG_223, and *ftsZ*. In a previous study, we demonstrated that *ftsZ* was dispensable for growth of *M. genitalium* under laboratory culture conditions. Herein, we show that the entire cell division gene cluster of *M. genitalium* is non-essential for growth *in vitro*. Our analyses indicate that loss of the *mraZ* gene alone is more detrimental for growth of *M. genitalium* than deletion of *ftsZ* or the entire cell division gene cluster. Transcriptional analysis revealed a marked upregulation of *ftsZ* in the *mraZ* mutant. Stable isotope labeling by amino acids in cell culture (SILAC)-based proteomics confirmed the overexpression of FtsZ in MraZ-deprived cells. Of note, we found that *ftsZ* expression was upregulated in non-adherent cells of *M. genitalium*, which arise spontaneously at relatively high rates. Single cell analysis using fluorescent markers showed that FtsZ localization varied throughout the cell cycle of *M. genitalium* in a coordinated manner with the chromosome and the terminal organelle (TMO). In addition, our results indicate a possible role for the RNA methyltransferase MraW in the regulation of FtsZ expression at the post-transcriptional level. Altogether, this study provides an extensive characterization of the cell division gene cluster of *M. genitalium* and demonstrates the existence of regulatory elements controlling FtsZ expression at the temporal and spatial level in mycoplasmas.

## Introduction

**Figure 1:**
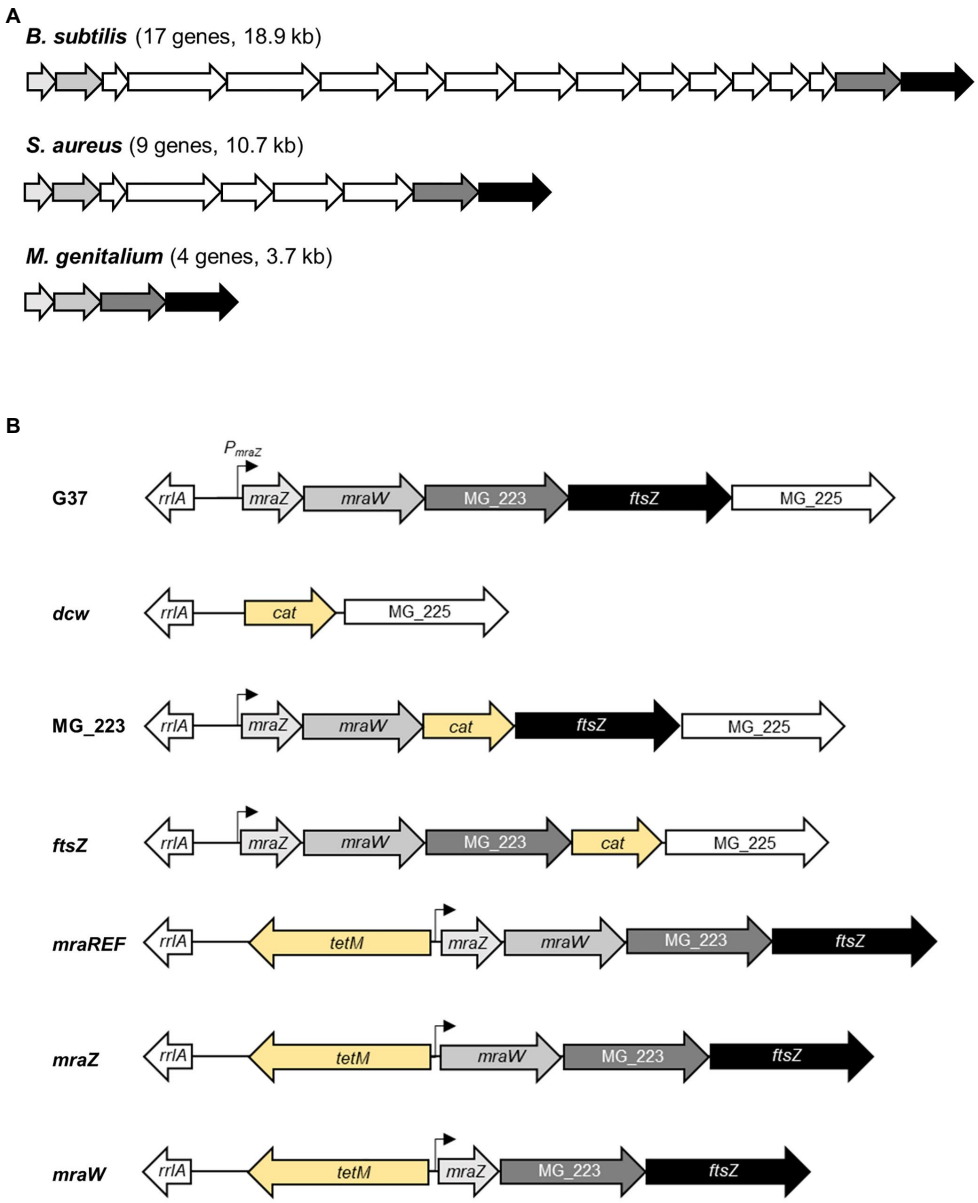
Overview of the *dcw* gene cluster organization in selected bacteria. Scheme depicting the *dcw* gene cluster of three representative species of the Firmicutes phylum **(A)**. *Bacillus subtilis* (17 genes, 18.9kb), *Staphylococcus aureus* (nine genes, 10.7kb), and *Mycoplasma genitalium* (four genes, 3.7kb). **(B)** Scheme of the cell division gene cluster in the different *M. genitalium* mutants created in this study. The promoter region of the *mraZ* gene (P_mraZ_) was characterized in a previous report (Benders et al., 2005). The *tetM* or *cat* markers (in yellow) were introduced to select for the intended mutants.

Cell division plays a central role in the life of all prokaryotic and eukaryotic organisms and it requires the coordinated action of multiple proteins and regulatory circuits. In bacteria, most genes necessary for cytokinesis and peptidoglycan wall biosynthesis are encoded in the division and cell wall (*dcw*) gene cluster. Organization and length of the *dcw* gene cluster are exquisitely conserved across phylogenetically distant species. As an exception, several genes that lie within the *dcw* gene cluster of Gram-negative and Gram-positive rods, are located elsewhere in the chromosome in Gram-positive cocci (Pucci et al., 1997). Based on this observation, a relationship between the structure of the *dcw* gene cluster and cell morphology was proposed. The underlying mechanism in this relationship, which involves the co-translational assembly of the protein complexes involved in cell division, is referred to as genomic channeling (Mingorance et al., 2004). In mycoplasmas, the *dcw* gene cluster is usually limited to four genes: *mraZ*, *mraW*, MG_223, and *ftsZ* (Figure 1A; Alarcón et al., 2007). Mycoplasmas are phylogenetically related to Gram-positive bacteria, but they have lost the peptidoglycan biosynthesis genes as a result of an extensive genome reduction. Although mycoplasma cells are typically spherical, the presence in some species of a tip structure instrumental for cytoadherence, results in an elongated, flask-shaped morphology.

The function of *mraZ* and *mraW*, the first two genes of the *dcw* gene cluster, has been elusive for many years. A comprehensive study in *Escherichia coli* revealed that MraZ is a transcriptional repressor that controls its own expression and that of other genes of the *dcw* gene cluster (Eraso et al., 2014). In the same study, the authors found that MraZ binds to conserved sequences, designated as MraZ boxes, within the upstream region of the *dcw* gene cluster. Perhaps surprisingly, loss of MraZ was not associated with any apparent phenotype in this model bacterium. In spite of this, an antagonistic effect between MraZ and MraW was disclosed. MraW is an RNA methyltransferase that targets the 16S ribosomal RNA (Kimura and Suzuki, 2010). Characterization of an *mraW* mutant in *E. coli* demonstrated an altered non-AUG initiation and a decreased translation fidelity, suggesting that MraW could play a role in start codon selection and recognition of classic STOP codons. In *Staphylococcus aureus*, *mraW* mutants also exhibit an anomalous translation fidelity, along with a reduced growth rate and an increased sensitivity to oxidative stress (Kyuma et al., 2015). More recently, the MraW protein was found to also methylate DNA and alter gene expression in *E. coli* (Xu et al., 2019). Of note, loss of MraW increases the expression of MraZ, which supports the antagonistic activity between these two proteins described earlier (Eraso et al., 2014; Xu et al., 2019).

By contrast, the function of the ancestral homologue of tubulin, FtsZ, is much better established. FtsZ polymerizes and depolymerizes through GTP hydrolysis and forms a ring-like structure at the midcell known as the Z-ring that subsequently contracts during septation (Adams and Errington, 2009; Busiek and Margolin, 2015). The Z-ring recruits cell division proteins and septal peptidoglycan synthesizing enzymes to the division site, and constitutes the scaffold of the bacterial divisome (Errington et al., 2003). In *E. coli*, FtsA is the first protein known to be recruited to the septal ring and it is important for the stability of FtsZ (Vicente and Rico, 2006). Of note, the ratio of FtsZ and FtsA is critical for the proper functioning of the cell division apparatus (Dai and Lutkenhaus, 1992; Dewar et al., 1992). In fact, it has been recently demonstrated that cell division in the Gram-positive bacterium *Bacillus subtilis* is mediated by filaments of FtsZ and FtsA, which treadmill circumferentially around the division ring, driving the motion of the peptidoglycan synthesizing enzymes (Bisson-Filho et al., 2017).

Because of its pivotal role in cell division, FtsZ expression is controlled at multiple levels (Robin et al., 1990). Similarly, septal ring formation and localization are tightly regulated (Vicente et al., 1998). The complex regulation observed within the *dcw* gene cluster has a fundamental biological role and accordingly, dissociation of *ftsZ* expression from its natural regulatory signals leads to important alterations in the physiology of cell division. However, so far, very little is known regarding the factors controlling FtsZ expression, localization, and function in mycoplasmas. The cell division gene cluster has an operonic structure in *Mycoplasma genitalium* and the close related species *Mycoplasma pneumoniae* (Benders et al., 2005). On the other hand, the N-terminal region of FtsZ from *Mycoplasma pulmonis* could function for cell division in *E. coli* suppressor strains, provided that the C-terminal tail is replaced (Osawa and Erickson, 2006). In addition, in *Mycoplasma hominis*, FtsZ protofilaments can form spiral structures similar to Z-spirals of *B. subtilis* and *E. coli*, suggesting also a functional role of FtsZ in cell division (Vishnyakov et al., 2009). Recent data also suggest that FtsZ stability is controlled by the Lon protease in *M. pneumoniae* (Burgos et al., 2020).

Remarkably, while the *ftsZ* gene is essential in most bacteria (Beall and Lutkenhaus, 1991; Dai and Lutkenhaus, 1991), it is dispensable in cell wall-deficient derivatives of some Gram-positive and Gram-negative bacteria known as L-forms (Mercier et al., 2014). Similarly, bacteria from the *Planctomycetes* and Chlamydiae superphylum lack the FtsZ protein (Pilhofer et al., 2008). In a previous study, our laboratory demonstrated that *ftsZ* was non-essential for *in vitro* growth of *M. genitalium* (Lluch-Senar et al., 2010), raising important questions as to the conservation of *ftsZ* in mycoplasmas. In keeping with this, other species phylogenetically related to *M. genitalium* such as *Mycoplasma mobile* or *Ureaplasma urealyticum*, do not code for a homolog of the *ftsZ* gene (Glass et al., 2000; Jaffe et al., 2004). Therefore, what is the role of the cell division genes in mycoplasmas? We have addressed this question by constructing and characterizing several cell division mutants in the genome-reduced bacterium *M. genitalium*.

## Materials and Methods

### Bacterial Strains and Culture Conditions

All *M. genitalium* strains were grown in SP-4 broth at 37°C in a 5% CO_2_ atmosphere in tissue culture flasks. SP-4 plates were prepared supplementing the medium with 0.8% agar (BD). Chloramphenicol (17μgml^−1^) or tetracycline (3μgml^−1^) were added for mutant selection. All *M. genitalium* strains used in this work are listed in the Supplementary Table S1. *Escherichia coli* strain XL-1 Blue was used for cloning and plasmid propagation. The strain was grown in Luria Bertani (LB) or LB agar plates containing 100μgml^−1^ ampicillin, 40μgml^−1^ X-Gal, and 24μgml^−1^ Isopropyl β-D-1-thiogalactopyranoside (IPTG) when needed.

### DNA Manipulation

Plasmid DNA was obtained using 5Prime kit. PCR products were purified from agarose gels using Nucleospin Gel and PCR Clean-up kit (Macherey-Nagel) and digested with the corresponding restriction enzymes (Fermentas) when necessary. All primers used in this study are listed in the Supplementary Table S2. Plasmids for *M. genitalium* transformation were obtained using the GenElute HP Midiprep kit (Sigma).

### Mutant Construction and Screening

A detailed explanation of the methodology, including primers and plasmids, used to generate the different mutants created in this study is supplied in the Supplementary Material. Integrity of the chromosome in the mutant strains, other than the intended deletions, was assessed and confirmed by whole genome sequencing (WGS; Supplementary Table S3). Transformation of *M. genitalium* was carried out as previously described (Torres-Puig et al., 2015). Screening for mutants was performed using cell lysates as template for PCR or sequencing reactions. Cell lysates were obtained by centrifugation of 1.5ml cell cultures, disruption of pellets using 30μl of Lysis Buffer (Tris-HCl 0.1M pH8.5, Tween-20 0.05%, and proteinase K 0.25mgml^−1^), and incubation for 1h at 37°C followed by inactivation at 95°C for 10min.

### DNA Sequencing

DNA sequencing reactions were performed using BigDye® v3.1 Cycle Sequencing kit using 2.5μl of genomic DNA or *M. genitalium* lysate, following manufacturer’s instructions. All reactions were analyzed in an ABI PRISM 3130xl Genetic Analyzer at the Servei de Genòmica i Bioinformàtica (UAB).

### Growth Rate Quantification

Growth rates of *M. genitalium* cultures were determined using an adaptation of the colorimetric protocol described by Karr et al. (2012). Strains were grown to mid-log phase in 25cm^2^ flasks with 5ml SP4. Attached cells were scraped off, recovered by centrifugation at 12,000rpm, and resuspended in 3ml of fresh SP4. Then, 300μl of the cellular suspension were inoculated in four different wells of a 96-well plate. Each of these wells constitutes the first sample of four technical repeats. About 100μl of the first well (sample 1) were inoculated into the next well (sample 2) already containing 200μl of fresh SP4, thus diluting 1/3 the initial concentration. This process was repeated four more times (samples 3, 4, 5, and 6), achieving a final dilution of 1/243. The 96-well plate was sealed with transparent tape, placed into a Tecan Sunrise Absorbance Microplate Reader (Tecan), and incubated at 37°C for 7days. As *M. genitalium* grows, the bacterial metabolism acidifies the culture medium changing the color of the phenol red indicator from red (pH 7.8) to yellow (pH 6), which was detected by measuring the absorbance at 550nm. For each analysis, readings were taken every 30min for up to 8days (total of 400 reads per well). The readings were stored in an Excel datasheet and analyzed once the experiment was completed, and a curve was plotted for each dilution and the inflection point was inferred. Next, the inflection points were plotted in a graphic, using the Napierian logarithm of the dilution as the *x* coordinate for each dilution. Once all the inflection points were plotted, the slope (*μ*, growth rate constant) was inferred by linear regression, and the doubling time (*g*) was obtained and according to the general equation for exponential growth of bacteria [*g*=ln2/(1/*μ*)].

### RNA-Seq

RNA-Seq analyses were performed as previously described (Torres-Puig et al., 2018; Martínez-Torró et al., 2020). Mid-log phase cultures of *M. genitalium* were scraped off in 1ml of fresh SP-4 and reinoculated in two new 25cm^2^ tissue culture flasks with fresh SP-4 medium for 6h. Then, cells were lysed, and total RNA was extracted using the miRNeasy Mini Kit (Qiagen). RNA libraries were prepared with TruSeq Stranded Total RNA Library Prep Kit (Illumina) and analyzed using a HiSeq 3000 System (Illumina) at the Genomics Unit from the Centre for Genomic Regulation (CRG), Barcelona. cDNA clusters were immobilized in sequencing lanes of 2×50 reads. Reverse and complementary were computed for sequences coming from Read1 primer. Data analysis and sequence alignment were performed using Bowtie2 (Langmead and Salzberg, 2012) in the End-to-End mode and Forward-Forward paired-ends. Sequences were piled up using SAMtools (Li et al., 2009) with no limited set to the number of sequences in the alignment. Counts in the different ORFs were performed with a standalone version of featureCounts program (Liao et al., 2014) without counting the multi-mapping reads and disabling multi-overlapping reads.

Counted features were then submitted to the R/Bioconductor package DESeq2 (Love et al., 2014) for statistical analysis. DESeq2 analysis used a parametric fitType and a zero-mean normal prior on the non-intercept coefficients. Data were sorted by log2 fold change, and statistical significance was set at the common threshold value of *p*<0.05. Three independent biological repeats of each strain or condition were analyzed.

### qRT-PCR

RNA was extracted from mid-log phase cultures of *M. genitalium* using the RNAqueous Kit (Thermo Fisher Scientific) and then treated with Turbo DNase (Thermo Fisher Scientific) following the manufacturer’s instructions. Reverse transcription was performed with iScript Reverse Transcriptase (Bio-Rad) and random primers as previously described (Torres-Puig et al., 2015). Primers used for qPCR are listed in Supplementary Table S2 and they were designed using Primer3 software. qPCR was performed with iTaq polymerase (Bio-Rad) and SYBR green in CFX96 or CFX384 PCR instruments (Bio-Rad). Relative gene expression was calculated using the Pfaffl method (Pfaffl, 2001). Differential gene expression was judged based on the common arbitrary 2-fold cutoff. Data presented in the manuscript correspond to the analysis of RNAs isolated from three independent biological repeats.

### Quantification of Protein Abundance

Differences in relative protein abundance were assessed by stable isotope labeling by amino acids in cell culture (SILAC). To this end, *M. genitalium* cultures were grown to mid-log phase in 25cm^2^ flasks on Hayflick’s medium with 15mM of light (^12^C) or heavy (^13^C) lysine. Once grown, cells were split (1:10) and cultured in the same conditions. Then, cells were washed with PBS, scraped off, and pellets stored at −80°C. Protein lysates were prepared as previously described (Sabidó et al., 2012). Protein quantification was performed using the Pierce BCA Protein Assay Kit (Thermo Fisher Scientific), and protein extracts from heavy and light media cultures were mixed at a 1:1 ratio. Samples were then reduced with dithiothreitol (150nmol, 1h, 37°C) and alkylated in the dark with iodoacetamide (300nmol, 30min, 25°C). The resulting protein extract was diluted 1/3 with 200mM NH_4_HCO_3_ and digested with 5μg LysC (Wako) overnight at 37°C. Finally, the peptide mix was acidified with formic acid and desalted with a homemade Empore C18 column (3M, St. Paul, MN, United States; Rappsilber et al., 2007). Samples were analyzed using an LTQ-Orbitrap Velos Pro mass spectrometer (Thermo Fisher Scientific, San Jose, CA, United States) coupled to an EasyLC [Thermo Fisher Scientific (Proxeon), Odense, Denmark]. Peptides were loaded directly onto the analytical 25-cm column with an inner diameter of 75μm and packed with 5-μm C18 particles (Nikkyo Technos Co. Ltd., Japan). Chromatographic gradients started at 97% buffer A and 3% buffer B, with a flow rate of 250nl/min, and gradually increased to 65% buffer A/35% buffer B over 360min. After each analysis, the column was washed for 10min with 10% buffer A/90% buffer B. Buffer A: 0.1% formic acid in water. Buffer B: 0.1% formic acid in acetonitrile. The mass spectrometer was operated in positive ionization mode with a nanospray voltage set at 2.2kV and source temperature at 250°C. Ultramark 1621 for the FT mass analyzer was used for external calibration prior to the analyses. Moreover, an internal calibration was also performed using the background polysiloxane ion signal at *m*/*z* 445.1200. The instrument was operated in data-dependent acquisition (DDA) mode and full MS scans with one micro scan at a resolution of 60,000 were used over a mass range of *m*/*z* 350–2,000 with detection in the Orbitrap. Auto gain control (AGC) was set to 10^6^, and dynamic exclusion (60s) and charge state filtering disqualifying singly charged peptides were carried out. In each cycle of DDA analysis, following each survey scan, the top 10 most intense ions with multiple charged ions above a threshold ion count of 5,000 were selected for fragmentation at normalized collision energy of 35%. Fragment ion spectra produced *via* collision-induced dissociation (CID) were acquired in the ion trap, AGC was set to 5e^4^, with an isolation window of 2.0 *m*/*z*, activation time of 0.1ms, and maximum injection time of 100ms. All data were acquired with Xcalibur software v2.2. The MaxQuant software suite (v1.4.0.5) was used for peptide identification and SILAC protein quantitation (Cox and Mann, 2008). The data were searched against an in-house generated database containing all *M. genitalium* proteins.

### Scanning Electron Microscopy

*Mycoplasma genitalium* cultures were grown to mid-log phase over glass coverslips. Samples were fixed, dehydrated, and critical point dried as previously described (Pich et al., 2008). Coverslips were then sputter coated with gold and examined in a Merlin (Zeiss) scanning electron microscope (SEM). SEM micrographs were analyzed with the ImageJ software to determine the frequency of cells in division. To this end, we determined the number of terminal organelles (TMOs) per cell. As mycoplasma cells duplicate the tip structure during cell division, cells with one TMO were classified as non-dividing, while cells with two or more TMOs were classified as dividing. On the other hand, we measured the length of single cells from the tip of the TMO to the opposite pole. In addition, we determined the length of cytokinetic cells by measuring the distance from the tip of one TMO to the tip of the opposite TMO. For this analysis, only cells with one TMO at each cell pole were considered.

### Phase Contrast and Fluorescence Microscopy

*Mycoplasma genitalium* cells were grown in filtered SP-4 medium (0.22μm) on IBIDI chamber slides for 16h, washed once with 1×PBS, and visualized on an inverted Nikon Eclipse TE 2000-E microscope. For the analysis of the mg191ftsZCh non-adherent mutant, IBIDI chamber slides were previously treated with 0.2mg/ml poly-l-lysine hydrobromide (Sigma-Aldrich) solution, allowing surface adherence. In addition, cells were passed 10 times through a 25-gauge syringe to break the aggregates. Hoechst 33342 0.01mgml^−1^ was added when necessary. All strains were grown and visualized under the same conditions. Phase contrast, enhanced yellow fluorescent protein (eYFP), 4',6-diamidino-2-phenylindole (DAPI), and TRITC epifluorescence images were captured with a Digital Sight DS-SMC Nikon camera controlled by NIS-Elements BR software. Images were analyzed using ImageJ software and GDSC plug-in.

## Results

### Deletion of the Entire Cell Division Gene Cluster of *Mycoplasma genitalium*

**Figure 2:**
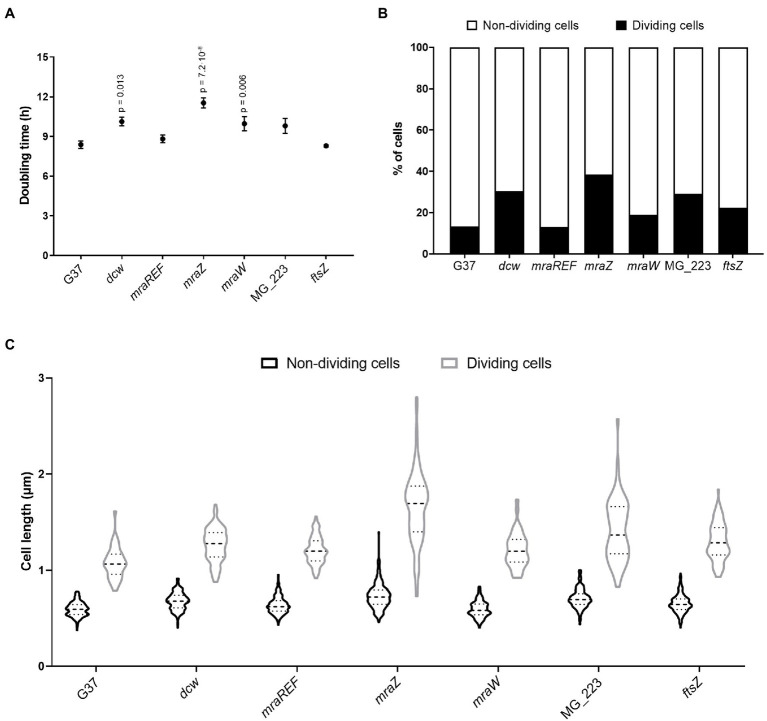
Cell fitness analysis of the wild-type strain and several *M. genitalium* mutants. Growth rates **(A)** and percentage of cells in division **(B)** of different *M. genitalium* mutants of the cell division gene cluster. Statistical significance was assessed using the Student’s *t*-test and the value of *p* of significant values (*p*<0.05) is indicated above each data point. An average of 900 cells for each strain were analyzed **(C)**. Graph depicting the length of single or cytokinetic cells. An average of 320 cells for each strain were measured.

Previously, we demonstrated that the *ftsZ* gene was non-essential in *M. genitalium* (Lluch-Senar et al., 2010). Based on this finding, we speculated whether the entire cell division gene cluster could be dispensable in this wall-less bacterium. To ascertain this, we replaced the four genes of the cell division gene cluster of *M. genitalium* by the chloramphenicol resistance marker (Figure 1B). We recovered several chloramphenicol resistant transformants and confirmed the deletion of the entire gene cluster (3.7kb) by PCR and sequencing. We found that the duplication time of the resulting *dcw* mutant was significantly longer (10.13±0.33h) than that of the wild-type strain (8.38±0.28h; Figure 2A). In addition, SEM analyses revealed an increased frequency of cells in division in the *dcw* mutant (30.53%) as compared to the wild-type strain (13.35%; Figure 2B).

### Characterization of Cell Division Mutants of *Mycoplasma genitalium*

To dissect and better understand the phenotype of the *dcw* mutant, we created strains defective for each gene of the cell division gene cluster by allelic exchange. First, we obtained mutants for the MG_223 and *ftsZ* genes using the chloramphenicol resistance marker for selection (Figure 1B; Supplementary Material). Our analyses revealed that the growth rate of the *ftsZ* mutant (8.29±0.09h) was comparable to that of the wild-type strain (8.38±0.28h; Figure 2A). By contrast, the duplication time of the MG_223 mutant increased significantly (9.80±0.56h). SEM analyses revealed an increased frequency of cells in division in both mutant strains (*ftsZ*, 22.44%; MG_223, 29.15%; Figure 2B).

Next, we created mutants lacking either *mraZ* or *mraW*. In this case, to preserve the operonic structure of the gene cluster, the antibiotic resistance marker was placed at the 5' end of the *mraZ* UTR (Figure 1B; Supplementary Material). For control purposes, we also created a reference strain, designated *mraREF*, carrying the selectable marker in the same chromosomal location, but with an intact cell division gene cluster (Figure 1B; Supplementary Material). Cells from the *mraREF* strain grew at a similar rate (8.81±0.29h) than those of the wild-type strain (8.38±0.28h; Figure 2A). However, the duplication time increased significantly upon the loss of MraZ (11.54±0.38h). Cells from the *mraW* null mutant duplicated at a slower rate (9.96±0.53h), indicating that the absence of MraW is also detrimental for growth. All the mutant strains obtained were examined by genome resequencing for the presence of mutations other than those introduced by genetic manipulation. No major genome rearrangements like large deletions or translocations were detected in all the analyzed strains. However, all the strains showed the presence of small variants like SNPs or INDELS in different frequencies. Many of these variants were also identified in the G37 strain. All new variants were found in recombinogenic, variable repeated regions, being most of them in non-coding genome locations, thus supporting the absence of additional mutations that could impact on the phenotype of the *dcw* strains (Supplementary Material).

**Figure 3:**
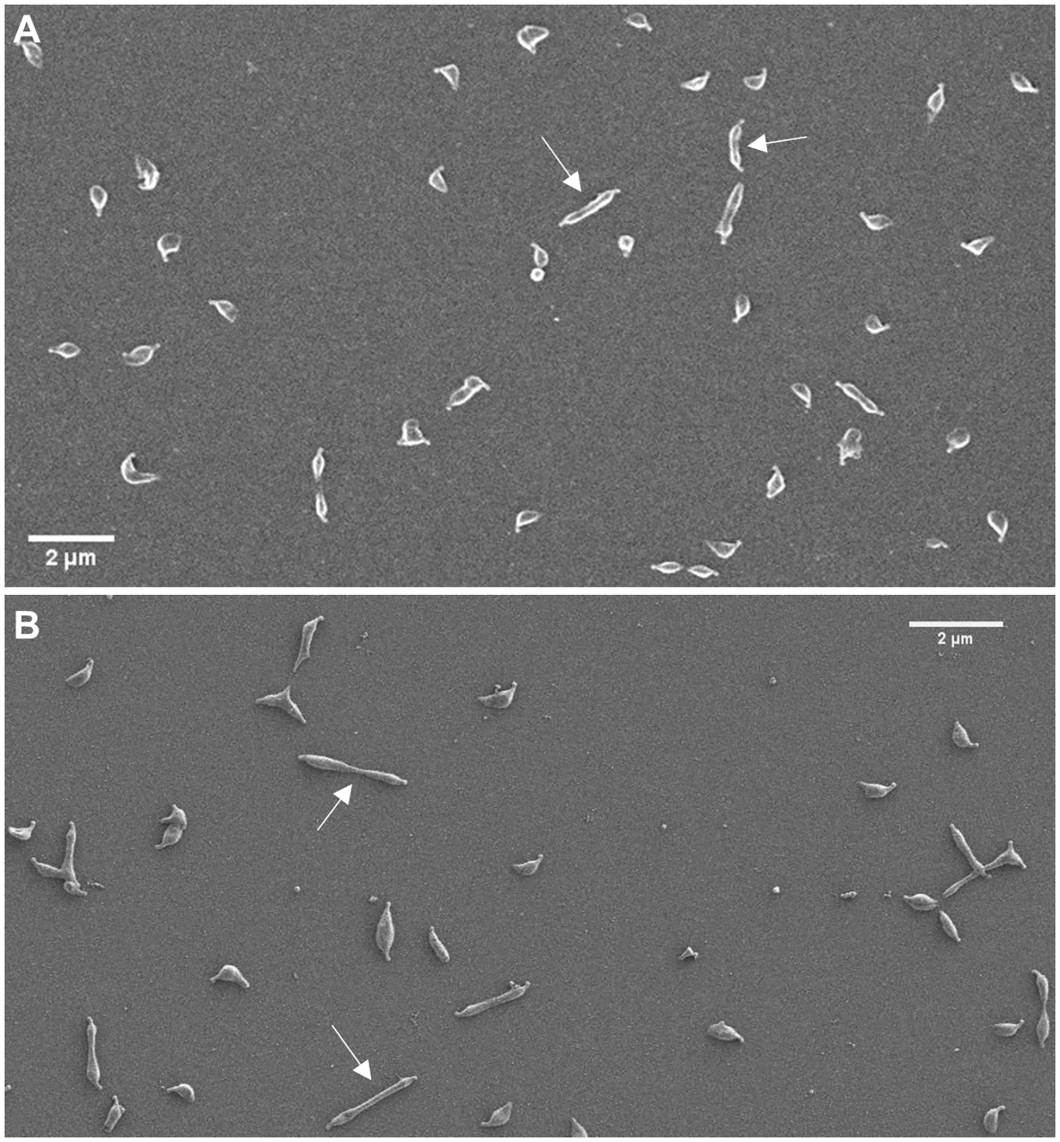
Characterization of cell morphology of the *mraZ* mutant. Scanning electron micrographs of the wild-type strain **(A)** and the *mraZ* mutant **(B)**. White arrows indicate some cells in cytokinesis.

Scanning electron microscopy analyses revealed a slight increase in the frequency of cells in division in the *mraW* mutant (19.0%) when compared to the wild-type (13.35%) or the *mraREF* strains (12.99%; Figure 2B). Remarkably, the frequency of dividing cells found in the *mraZ* mutant was markedly high (38.44%). Moreover, we observed that cytokinetic cells from the *mraZ* mutant were markedly elongated (Figures 3A,B). Quantitative analysis confirmed that cells in cytokinesis from the *mraZ* mutant were longer (1.651±0.372μm, *n*=96) than those of the wild-type strain (1.118±0.177μm, *n*=24; Figure 2C). By contrast, analyses of the other cell division mutants obtained in this study did not reveal morphological changes in the cytokinetic cells (Figure 2C).

### Transcriptional Analysis of the Cell Division Gene Cluster in Different Mutant Backgrounds

**Figure 4:**
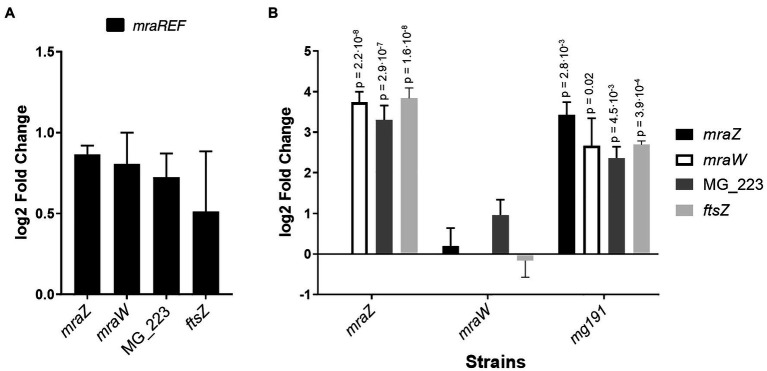
Transcriptional analysis of the cell division gene cluster of several *M. genitalium* mutants by qRT-PCR. Transcriptional changes of the cell division gene cluster in the *mraREF* mutant **(A)** or the *mraZ*, *mraW*, and *mg191* mutants **(B)** compared to the wild-type strain. Bars represent the average log2 fold change of at least three independent biological repeats. Statistical significance was assessed using the Student’s *t*-test, and the value of *p* of biologically significant values (log2±1) is stated above the corresponding bar if significant (value of *p*<0.05).

Previous studies in *E. coli* indicated that MraZ is a transcriptional repressor (Eraso et al., 2014). Therefore, we conducted a genome-wide transcriptional analysis in *M. genitalium* by RNA-Seq to identify transcriptional changes in the *mraZ* mutant (Table 1). Our transcriptomic data revealed that *mraW*, *ftsZ*, and the MG_223 gene were significantly upregulated in the absence of MraZ. By contrast, the transcription was largely unaffected in both the *mraW* mutant and the *mraREF* control strain. qRT-PCR analyses confirmed the increased expression of the cell division genes in the absence of MraZ (Figure 4). To confirm that the altered gene expression observed in the *mraZ* mutant was due to the loss of the MraZ protein, we reintroduced an ectopic copy of the *mraZ* gene under the control of its native promoter. Of note, several complementation attempts with a transposon encoded copy of the *mraZ* gene, alone or in combination with *mraW*, were unsuccessful. However, insertion of the *mraZ* gene at the end of the cell division gene cluster in the *mraZ* mutant restored the transcriptional levels of *mraW*, *ftsZ*, and the MG_223 gene (Figure 4; Supplementary Figure S1). On the other hand, we also assessed transcription of the cell division gene cluster in a mutant strain lacking the *mg191* gene, which codes for the major cytoadhesin P140 of *M. genitalium*. Cells from the *mg191* mutant are pleomorphic and grow in suspension as large cell aggregates (Burgos et al., 2006). This non-adherent mutant was analyzed to shed some light on the connection between cell division and adherence in *M. genitalium*. In this sense, in a previous study (Lluch-Senar et al., 2010), we showed that, in contrast to WT cells, an *ftsZ* mutant could not generate non-adherent variants. Based on this observation, we hypothesized that non-adherent mutants rely exclusively on the cell division cluster for division. Remarkably, transcriptional analysis of the *mg191* mutant revealed a significant upregulation of the cell division gene cluster (Figure 4).

**Table 1:** Transcriptional changes in the *mraZ* and *mraW* mutants by RNA-Seq.

Gene	*mraZ*		*mraW*	
	Log2 fold change	*p*	Log2 fold change	*p*
*mraZ*	**−4.64**	**0**	0.10	0.24
*mraW*	**3.02**	**0**	**−3.84**	**0**
MG_223	**2.08**	**2×10** ^**−139**^	−0.21	0.02
*ftsZ*	**2.34**	**9.3×10** ^**−218**^	−0.21	0.03

Transcriptional changes in the cell division gene cluster identified by RNA-Seq in the *mraZ* and *mraW* mutants. Changes that are statistically significant (value of *p*<0.05) and biologically relevant (above or below the log2±1 arbitrary cutoff) are highlighted in bold. Data were obtained from the analysis of three independent biological repeats of each mutant.

### Proteome Analysis of the *mraZ* and *mraW* Mutants

Next, we wondered whether the transcriptional changes observed in the *mraZ* null mutant were conserved at the protein level. To this end, we determined the proteome profile of the *mraZ* mutant by SILAC (Supplementary Tables S4 and S5). Our analyses demonstrated a marked increase in the cellular levels of MraW (14.3-fold) and FtsZ (25-fold; Table 2). Along the same lines, expression of the MG_223 protein could only be detected when MraZ was absent.

**Table 2:** Comparative analysis of the abundance of cell division proteins in the wild-type strain and the *mraZ* and *mraW* mutants by stable isotope labeling by amino acids in cell culture (SILAC).

Locus tag	Gene	Gene product	G37	mraZ		mraW	
			AUC	AUC	FC	AUC	FC
MG_221	*mraZ*	Transcriptional regulator MraZ	4.12×10^8^	ND	–	4.65×10^8^	–
MG_222	*mraW*	Ribosomal RNA small subunit methyltransferase H	4.17×10^7^	5.95×10^8^	**14.28**	ND	–
MG_223	–	Uncharacterized protein	ND	7.88×10^6^	–	ND	–
MG_224	*ftsZ*	Cell division protein	4.40×10^6^	1.10×10^8^	**24.96**	8.50×10^6^	1.93

Area under the curve (AUC) of the four proteins encoded in the cell division gene cluster in the wild-type (G37) and the *mraZ* and *mraW* mutants. Fold-change (FC) with respect to the G37 strain is indicated. Biologically significant fold changes (>2 and <0.5) are highlighted in bold. ND stands for not detected.

We also investigated the presence of proteomic changes in the *mraW* mutant (Supplementary Tables S6 and S7). We found that several proteins, including a number of DNA and RNA methyltransferases, were differentially expressed in the absence of MraW. As these changes in protein abundance were not accompanied by changes in mRNA levels, we anticipate that MraW may modulate protein expression at the post-transcriptional level. Of note, we found that the levels of the FtsZ protein were slightly higher (by 2-fold) in the *mraW* mutant (Table 2).

### FtsZ Expression and Localization Dynamics

**Figure 5:**
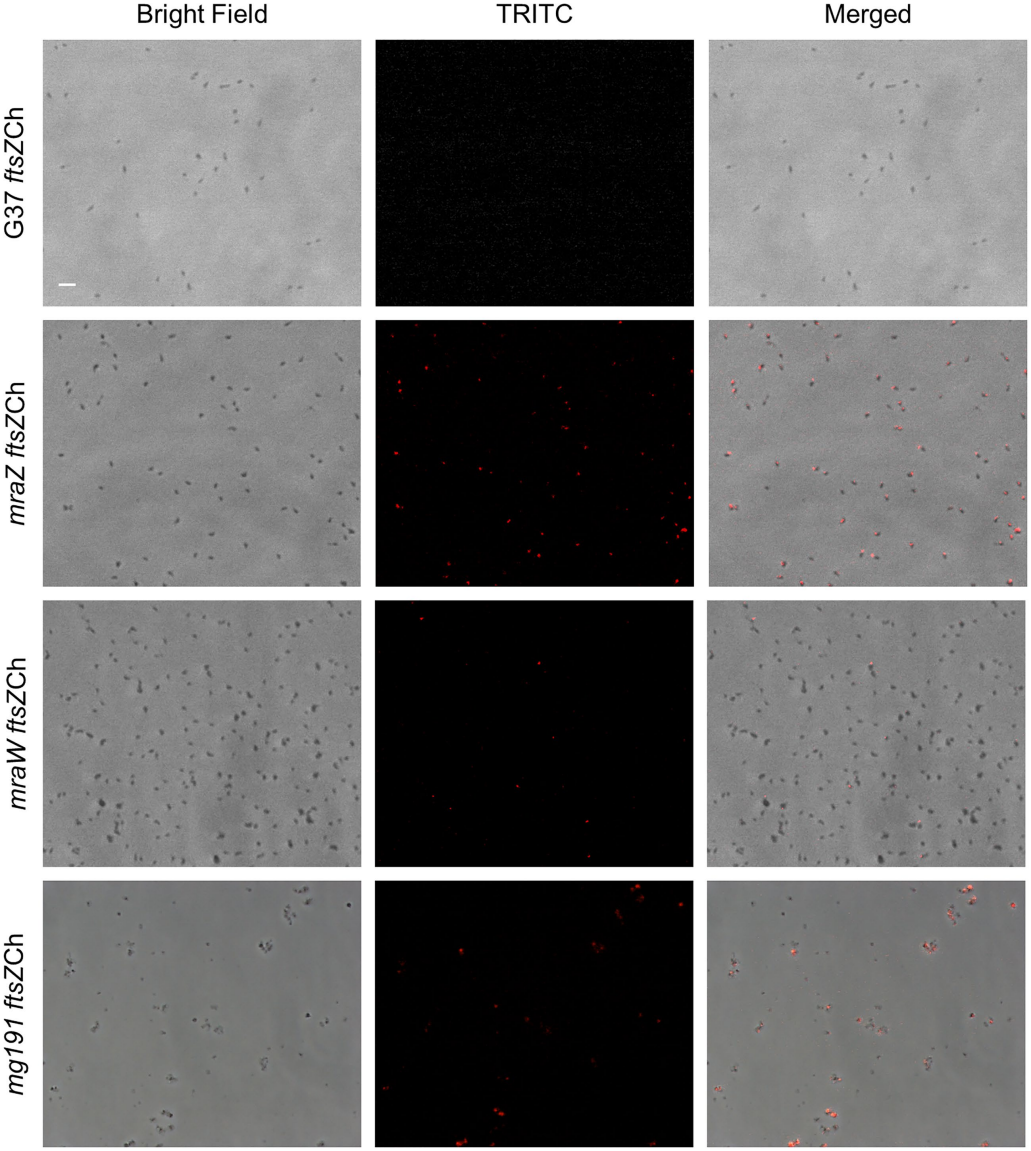
Single cell analysis of FtsZ expression in different *M. genitalium* mutants. Fluorescence microscopy images showing FtsZ-mCherry localization in live cells from the wild-type strain, *mraZ*, *mraW*, and *mg191* mutants. Each row contains the phase contrast, TRITC channel, and the resulting overlay, respectively. The scale bar represents 2μm. All images are shown at the same magnification.

Previous attempts in our laboratory to monitor FtsZ expression in *M. genitalium* using fluorescent markers were unsuccessful. In these studies, we characterized mutant strains carrying an *ftsZ*-*mcherry* fusion at its native locus, that is, at the 3' end of the cell division gene cluster (Figure 5). Presumably, FtsZ expression was too low to allow detection of the fluorescent fusion in single cells. However, we hypothesized that the increased levels of FtsZ expression observed in the *mraZ* mutant could facilitate the visualization of this protein in live cells. Therefore, we assessed FtsZ-mCherry expression in MraZ-deprived cells (Supplementary Material) and found a large number (56.63%) of fluorescent cells, although at different extents (Figure 5). The presence of non-fluorescent cells suggests that the expression and assembly of FtsZ are under the control of other factors in addition to MraZ (Burgos et al., 2020). Separately, we assessed FtsZ expression in the absence of MraW and observed that a small subset of cells (1.58%) exhibited mCherry fluorescence (Figure 5). This result is in keeping with the increased amount of FtsZ identified in the *mraW* mutant by proteomics analysis. On the other hand, we tested whether the FtsZ-mCherry fusion was visible in the P140 adhesin mutant. In agreement with the transcriptional data, we observed that some cells exhibited a conspicuous FtsZ-associated fluorescence (Figure 5).

**Figure 6:**
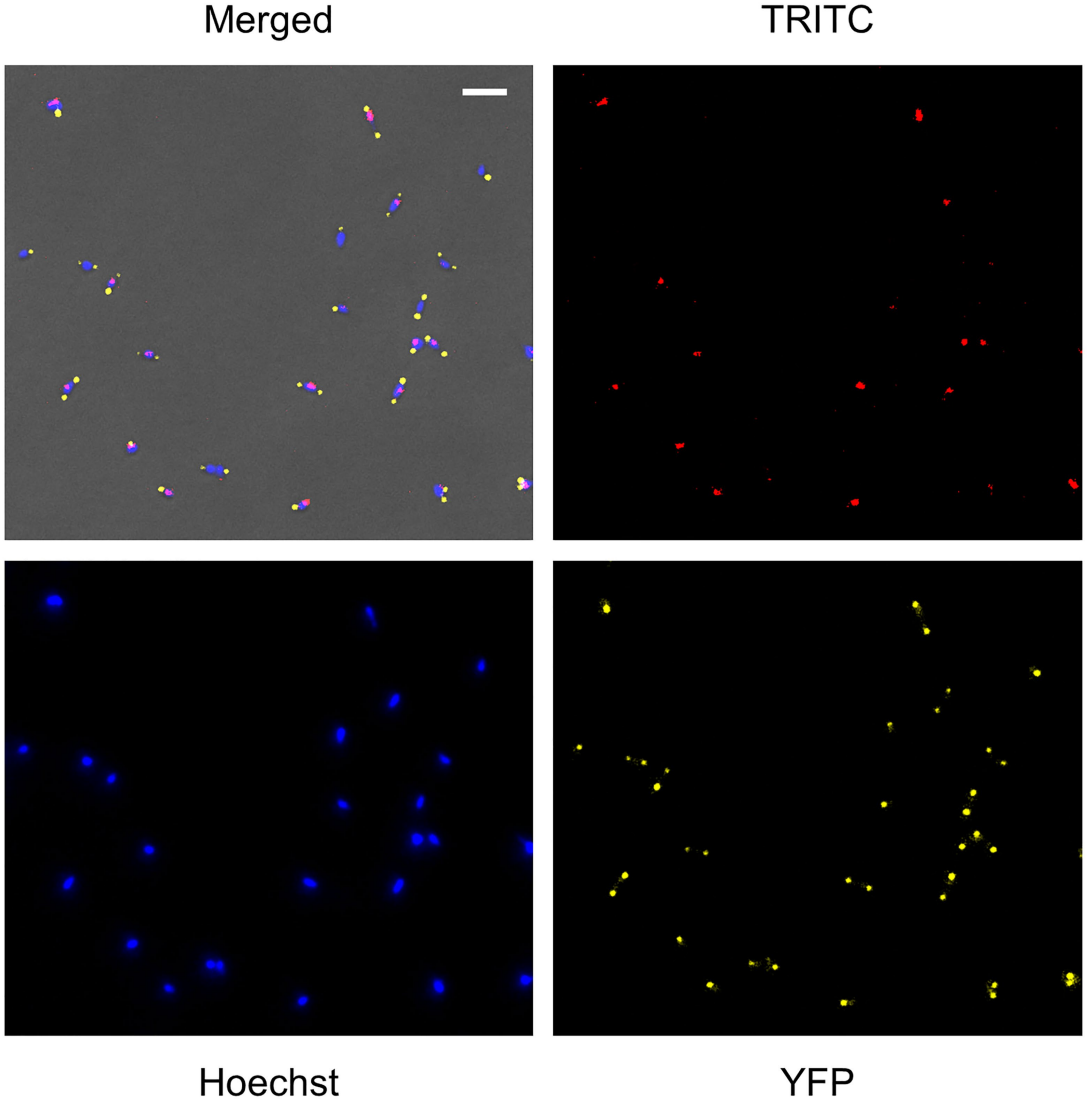
Single cell analysis of FtsZ, P65, and chromosome localization in the *mraZ* mutant. Fluorescence microscopy analysis showing FtsZ-mCherry, P65-eYFP, and chromosome localization in live cells of *M. genitalium*. Each panel shows the TRITC, 4',6-diamidino-2-phenylindole (DAPI), and eYFP images or a combination of the three channels and the phase contrast. The scale bar represents 2μm. All images are shown at the same magnification.

**Figure 7:**
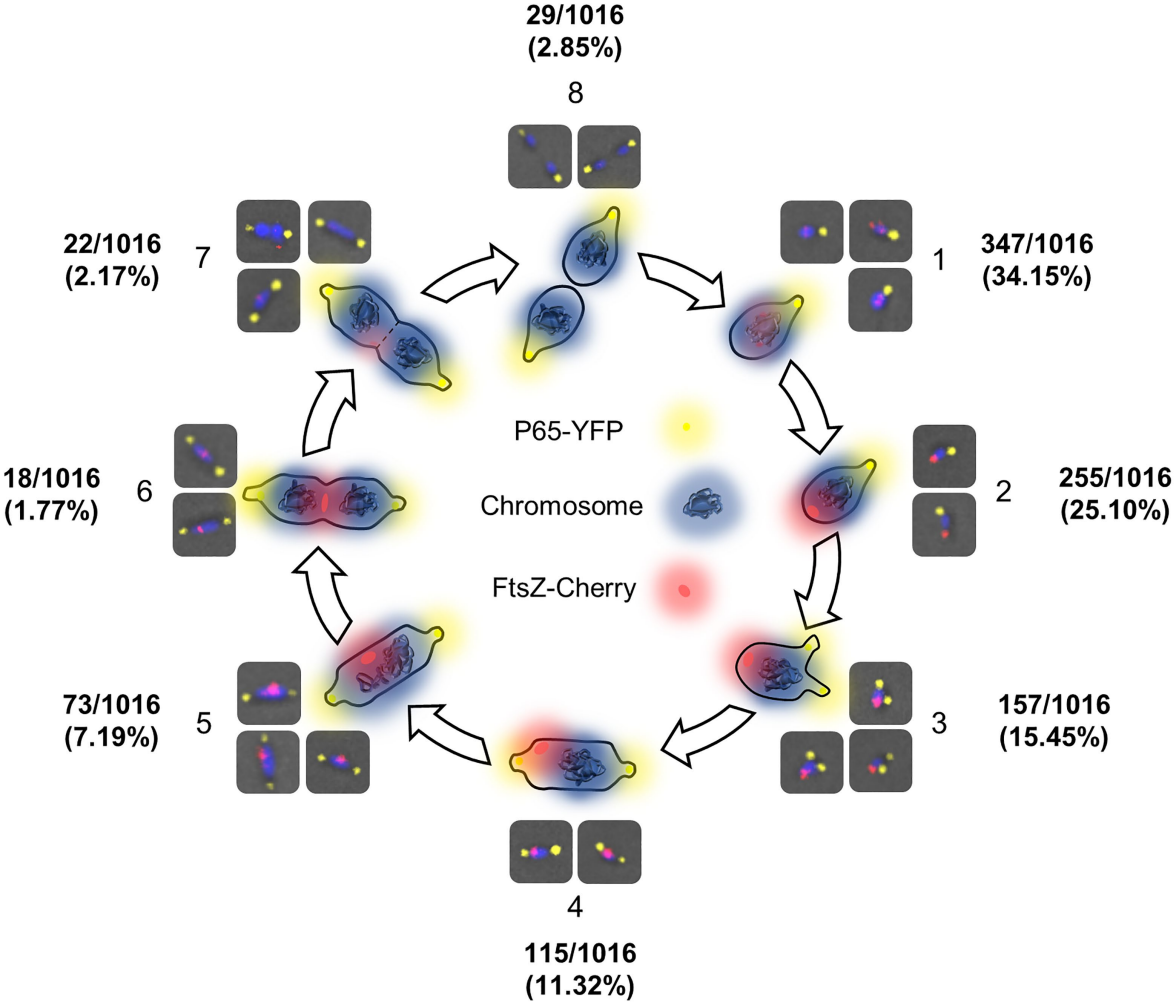
FtsZ localization dynamics throughout the cell cycle of *M. genitalium*. Scheme depicting different stages of the *M. genitalium* cell cycle based on the localization of FtsZ foci relative to the terminal organelle (TMO) and the chromosome. In this model, FtsZ accumulates at the cell body (1) and clusters at the cell pole opposite to the TMO (2). Then, the TMO duplicates (3) and one TMO moves to the opposite cell pole (4). Next, FtsZ foci move to the midcell (5). Finally, cytokinesis starts (6) and the FtsZ signal decreases (7) until the process is completed (8).

A detailed analysis of the *mraZ* mutant revealed that FtsZ foci displayed a polar location. Of note, *M. genitalium* cells are polar because they exhibit a TMO instrumental for cytoadherence. For this reason, we wanted to test whether FtsZ foci co-localized with the TMO of this bacterium. To this end, we tagged P65, a protein that localizes at the distal end of the TMO (Burgos et al., 2008). Introduction of an eYFP fluorescent marker at the P65 native locus (MG_217) allowed the identification of sharp P65-eYFP foci, providing a way to unambiguously identify the cell pole containing the TMO (Figure 6). We found that FtsZ clustered at the cell pole opposite to P65 in cells showing a single P65 focus. This is important because as mycoplasma cells duplicate the TMO prior to cytokinesis (Miyata and Seto, 1999), some cells displayed two separate P65 foci. In addition, we observed that the intensity and localization of the FtsZ foci varied through the cell division cycle. Based on the localization of the two tagged proteins (FtsZ and P65) and the chromosome stained with Hoechst 33258, we recognized eight different stages throughout the cell cycle (Figure 7). Non-dividing cells (stage 1) exhibit a single P65 focus and FtsZ signal is weak. In time, FtsZ is recruited at the cell pole opposite to the TMO (stage 2). Prior to cytokinesis, a new P65 focus forms close to the pre-existent focus (stage 3). Then, one of these P65 foci migrates to the opposite cell pole and localizes near the FtsZ focus (stage 4). Next, likely coinciding with chromosome segregation, FtsZ leaves the polar location and moves to the midcell to form a diffuse band (stage 5). As cytokinesis proceeds, FtsZ signal decreases (stage 6) and daughter cells display small FtsZ foci with marginal fluorescence (stage 7). Finally, daughter cells separate and FtsZ signal disappears (stage 8), but soon after, FtsZ accumulation starts within the cell body (stage 1).

## Discussion

Mycoplasmas are wall-less bacteria that divide by binary fission. So far, the factors mediating and coordinating cell division in these genome-reduced bacteria are poorly understood. Typically, the cell division gene cluster of mycoplasmas comprises four genes, including *ftsZ*. In walled bacteria, it is well-established that FtsZ drives peptidoglycan synthesis at the division site (Bisson-Filho et al., 2017). However, the conservation in mycoplasmas of FtsZ and other cell division related proteins, such as MraZ or MraW, is puzzling. In this study, we constructed and characterized several cell division mutants of *M. genitalium*. We found that deletion of *mraZ* induces a strong activation of the other genes of the cell division gene cluster. Hence, our results indicate that MraZ functions as a transcriptional repressor. This finding correlates well with proteomics data showing that MraW, FtsZ, and MG_223 proteins are overexpressed in the absence of MraZ. Altogether, our results substantiate the repressor role of MraZ previously documented by Eraso et al. (2014). Unlike in *E. coli*, MraZ depletion in *M. genitalium* results in a clear phenotype. The *mraZ* mutant exhibits a significant growth delay, important morphological alterations, and a high frequency of cells stuck in cytokinesis. These data indicate that expression of the cell division proteins of *M. genitalium* at relative high levels throughout the cell cycle is largely detrimental.

A previous study in *Mycoplasma gallisepticum* revealed that MraZ overexpression induces a characteristic cell filamentation (Fisunov et al., 2016), which is reminiscent to the phenotype described in the *mraZ* mutant of *M. genitalium*. Moreover, the filamentation described in *M. gallisepticum* was also associated with increased levels of FtsZ. In the same study, the authors define a binding site for MraZ in Mollicutes, characterized by a series of direct repeats of the sequence AAAGTG[T/G]. The only occurrence of this motif in the chromosome of *M. genitalium* lays within the promoter region of the *mraZ* gene. This finding is consistent with the lack of transcriptional changes outside the cell division gene cluster in our *mraZ* mutant. Of note, complementation of the *mraZ* mutant of *M. genitalium* could only be achieved in *cis*, that is, reintroducing a copy of the *mraZ* gene at an ectopic site within the cell division gene cluster. This result reveals a complex interplay between the relative location of the *mraZ* gene in the chromosome and MraZ activity. In this sense, our results indicate that the repressor role of MraZ requires a transcriptional linkage between the *mraZ* gene, the MraZ operator, and the cell division gene cluster.

On the other hand, we found that cultures of the *mraW* mutant grew at a slower rate than those of the wild-type strain. More importantly, we could detect FtsZ-mCherry fluorescence in a small subset of cells from this mutant. This fact is relevant because we could not detect FtsZ-mCherry expression in the wild-type background. Thus, MraW may play a role in regulating FtsZ expression in *M. genitalium*. This regulation must take place at the post-transcriptional level, as no transcriptional changes could be detected in the *mraW* mutant. In agreement with these data, our proteomics analysis revealed a slight increase of FtsZ levels in the absence of MraW. Of note, FtsZ expression in the *mraW* mutant is expected to be low because of the repressor activity of MraZ. The differential expression of several methyltransferases in the *mraW* mutant was also noticeable, which may represent a response of *M. genitalium* to the loss of the methyltransferase activity of MraW. The existence of species with orphan *mraW* genes supports a role for MraW independent from MraZ function in the regulation of cell division. It has been reported that MraW methylates the C1402 residue of the 16S rRNA (Kimura and Suzuki, 2010). This residue is involved in translation initiation and its mutation in *E. coli* leads to an increased doubling time (Jemiolo et al., 1985). Moreover, the loss of MraW function has been directly related to an altered translation fidelity (Kyuma et al., 2015), DNA methylation, and regulation of gene expression (Xu et al., 2019). Our RNA-Seq data from the *mraW* mutant suggest that MraW does not regulate gene expression in *M. genitalium*. However, whether MraW has a role in DNA or rRNA methylation in this bacterium still needs to be fully addressed.

The increased levels of FtsZ expression in the *mraZ* mutant allowed us to visualize this protein in single cells and to determine the FtsZ dynamics throughout the cell cycle of *M. genitalium*. We found that FtsZ clusters at the cell pole opposite to the TMO. When TMOs duplicate and migrate to the opposite cell pole, FtsZ-associated foci move toward the midcell. Intensity of the FtsZ foci decreases when they arrive at the midcell and the two chromosomes start to segregate. This pattern strongly suggests that FtsZ might play an active role in cell division and facilitates cytokinesis in *M. genitalium*. In addition, the localization of FtsZ protein during the cell cycle seems to be tightly regulated. Although the presence of a Min system (Lutkenhaus, 2007; Rowlett and Margolin, 2015) has not been described in mycoplasmas, it is presumed that unidentified regulatory factors inhibit the formation of a Z-ring close to the TMO. Interestingly, *M. genitalium* codes for a DivIVA domain-containing protein (MG211), a key component of the Min system of *B. subtilis* (Edwards and Errington, 1997). In addition, we observed that FtsZ foci were sometimes located between two DNA foci, suggesting that nucleoid occlusion is also in place in *M. genitalium*. Altogether, the distinct localization of the FtsZ protein throughout the cell cycle suggests the existence of unknown factors regulating cell division in this genome-reduced bacterium. These underlying regulatory mechanisms coordinate the cytokinesis process with chromosome segregation (Seto and Miyata, 1999).

The results of our study indicate that the cell division gene cluster of *M. genitalium* plays a minor role during *in vitro* propagation of wild-type cells. This is consistent with previous reports showing that motility seems to be the primary force facilitating cytokinesis in *M. pneumoniae*, a close relative species (Hasselbring et al., 2006). A few years ago, we documented that deletion of the *ftsZ* gene abrogated the occurrence of non-adherent phase variants in *M. genitalium* (Lluch-Senar et al., 2010). These non-adherent variants arise at relatively high frequencies and may play an important role to evade the immune response during infection (Burgos et al., 2018). Based on this observation, we proposed that FtsZ could be important for cell division in non-adherent cells of *M. genitalium*. Of note, non-adherent derivatives of *M. genitalium* are non-motile, largely pleomorphic and grow as large cell aggregates (Burgos et al., 2006). In the current study, we provide evidence that the cell division gene cluster is upregulated in a non-adherent mutant of *M. genitalium*. This finding is in agreement with our previous hypothesis that FtsZ and the formation of a rudimentary divisome may be required when gliding motility is unfeasible. Therefore, the strict parasitic lifestyle of *M. genitalium* and the intricate strategy to escape immune surveillance, likely imposes the conservation of the cell division gene cluster in this wall-less bacterium. Remarkably, most *Mycoplasma* species are non-motile, which may also enforce the conservation of the cell division genes. Supporting this notion, a recent report has demonstrated that *ftsZ* is important to restore cell morphology and proficiency of cell division in synthetic minimal cells (Pelletier et al., 2021).

## Data Availability Statement

The original contributions presented in the study are publicly available. This data can be found here: National Center for Biotechnology Information (NCBI) BioProject database under accession number PRJNA750275.

## Author Contributions

CM-T, ST-P, ML-S, LS, EQ, JP, and OQP contributed to conception and design of the study. CM-T, ST-P, MM-S, MH-R, CM-N, ML-S, and OQP performed the experiments. CM-T and ST-P performed the statistical analysis. CM-T and OQP wrote the first draft of the manuscript. ST-P, EQ, and JP wrote some parts of the manuscript. All authors contributed to the article and approved the submitted version.

## Funding

This work was supported by the grant BIO2017-84166-R from the Ministerio de Ciencia, Innovación y Universidades.

## Conflict of Interest

The authors declare that the research was conducted in the absence of any commercial or financial relationships that could be construed as a potential conflict of interest.

## Publisher’s Note

All claims expressed in this article are solely those of the authors and do not necessarily represent those of their affiliated organizations, or those of the publisher, the editors and the reviewers. Any product that may be evaluated in this article, or claim that may be made by its manufacturer, is not guaranteed or endorsed by the publisher.

## Abbreviations

SEM, Scanning electron microscopy
TMO, Terminal organelle
WGS, Whole genome sequencing
