# Supplementary material for: Functional Characterization of the Cell Division Gene Cluster of the Wall-less Bacterium Mycoplasma genitalium
Source: Front Microbiol. 2021 Sep 13;12:695572. doi: 10.3389/fmicb.2021.695572 (PMC8475190; doi:10.3389/fmicb.2021.695572)
Supplement: Supplementary file 1 [file Data_Sheet_1.PDF]

Construction of *M. genitalium* mutants

pmraZWTcTer. This suicide plasmid was used to create a strain carrying the tetracycline resistance marker in the same location as the *mraZ* and *mraW* mutants, for control purposes. The upstream region of *mraZ* was amplified by PCR with the oligonucleotides *mraZ Upstream F* and *mraZ Upstream R*. The downstream region was amplified using *mraW Downstream F* and *mraW Downstream R* as primers. The resistance marker with the transcription terminator at its 3' end was amplified from the pMG_236 plasmid[1] using the *TetTer-5'* and *TetTer-3'* oligonucleotides. These oligonucleotides had overlapping regions with the *mraZ* promoter and the *mraZ* upstream region, respectively, to allow the total amplification of the different fragments by SOE-PCR. The second fragment was amplified with the *mraZProm (Dw-F)* and *mraW Downstream R* oligonucleotides. The upstream region was put together with the marker using the *mraZ Upstream F* and *TetTer-5'* primers and the downstream region was merged with the antibiotic resistance gene using the *TetTer-3'* and *mraW Downstream R* oligonucleotides. Both regions were later amplified together with *mraZ Upstream F* and *mraW Downstream R* as primers. The whole amplicon was cloned into an *EcoRV*-digested pBE plasmid[2] and transformed into *M. genitalium*. The terminator sequence is present between the metal acquisition operon (MG_304-MG_302) and the *dnaK* gene (MG_305) and was identified using the TransTermHP software[3].

pΔmraZ. This plasmid was used to generate a *M. genitalium mraZ* null mutant by homologous recombination (HR). It was constructed similarly to *pmraZWTcTer*. It contained the same upstream and downstream regions, but it lacked the *mraZ* ORF. The promoter region was just upstream from *mraW* thanks to a large oligonucleotide (mraW Prom-F) that annealed with the 5' of *mraW* in its 3' end and contained an 80+ nucleotide long 5' tail that annealed with the immediate upstream region of the mraZ gene. Thus, the putative regulatory region was preserved to ensure that the possible phenotypical effects associated with the strain were directly related to the loss of *mraZ*. The upstream region and the tetracycline resistance marker were amplified as stated in the *pmraZWTcTer* plasmid (amplified using the *mraZ Upstream F* and *TetTer-5'* oligonucleotides). The *mraW* gene and the downstream region were amplified using the *mraWProm-F* and *mraW Downstream R* primers. Then, the two fragments could be

merged because of the sequence overlap created by the *TetTer-5'* oligonucleotide, which contained a tail complementary to the regulatory region of *mraZ* that was present in the second fragment because of the *mraWProm-F* oligonucleotide. Thus, the final product was amplified using again the *mraZ Upstream F* and *mraW Downstream R* oligonucleotides. This was cloned into an *EcoRV*-digested pBE and transformed into *M. genitalium*.

pΔmraW. This plasmid was used to generate a *M. genitalium mraW* null mutant by homologous recombination (HR). The upstream region and the tetracycline resistance marker were amplified as described above. As for the *mraZ* and downstream fragment, an oligonucleotide (*mraZ-R*) containing the 3' end of *mraZ* and a tail complementary to the 5' end of the MG_223 gene was used. So, in order to put the MG_223 gene just downstream of the *mraZ* stop codon, the mraZ ORF and its promoter region were amplified with the *mraZProm (Dw-F)* and *mraZ-R* oligonucleotides; and 1 kb of the MG_223 gene was amplified as usually with the *mraW Downstream F* and *mraW Downstream R* primers. Then, as the 3' end of the mraZ product was complementary to the 5' of the MG_223 fragment, both fragments were merged using the *mraZProm (Dw-F)* and *mraW Downstream R* oligonucleotides. As for the upstream part, it was amplified using the *mraZ Upstream F* and *mraZ-R* oligonucleotides, as the *TetTer-5'* oligonucleotide had a tail complementary to the regulatory region of *mraZ*, as stated previously. Finally, the whole fragment was put together with the *mraZ Upstream F* and *mraW Downstream R* primers. Then, it was cloned into an *Eco*RV-digested pBE and transformed into the *M. genitalium*.

pdcw. This construction was used to delete the whole division and cell wall operon of *M. genitalium*. The *mraZ* upstream region was amplified using the mraZ upstream F and mraZ upstream R primers. The downstream region was amplified using the *mg224-Dw-F* and *mg224-Dw-R(Cm)* oligonucleotides. The 3' of the first fragment and the 5' of the second fragment overlapped with the 5' and 3' of the *cat* marker, respectively. The chloramphenicol resistance was amplified using the *Cm-F* and *Cm-R* primers. Then, the three fragments were fused together with SOE-PCR. As the first fragment (mraZ upstream region) ends at 80 bp of the mraZ ORF, the *mraZ* boxes were not present in

this construction. The whole fragment was cloned into an *Eco*RV-digested pBE and later transformed into the G37 strain.

pMG223. This construction was aimed to obtain a MG_223 null mutant by HR. The upstream region was amplified using *MG223 (Up-F)* and *MG223 (Up-R)* primers. The downstream region was amplified with another pair of oligonucleotides: *MG223 (Dw-F)* and *MG223 (Dw-R)*. *MG223 (Up-R)* and *MG223 (Dw-F)* had an overhang end at their 3' and 5' ends, respectively, that overlapped with the 5' and 3' ends of the *cat* resistance. The chloramphenicol resistance was amplified using the *Cm-F* and *Cm-R* primers and then the three fragments were fused together using SOE-PCR. The fragment was cloned into an *Eco*RV-digested pBE and later transformed into the G37 strain.

pftsZ. This construction was aimed to obtain a single knockout of the ftsZ gene. The upstream and downstream regions of *ftsZ* were amplified using two pairs of oligonucleotides: *mraW(Dw-F)* and *223p438(XhoI)-R* for the upstream region and *mg224-Dw-F* and *mg224-Dw-R(Cm)* for the downstream fragment. The *Cm*R was amplified with the *Cm-F* and *Cm-R* primers, as already described. The 3' of the first fragment and the 5' of the second fragment overlapped with the 5' and 3' of the *cat* resistance, respectively. Thus, all three fragments were joined together with SOE-PCR and cloned into an *Eco*RV-digested pBE. This construction was transformed into the G37 strain.

pftsZCh. This construction was created to fuse a fluorescent reporter to FtsZ by HR. The *ftsZ* gene was amplified with the *mg224-F(cherry)* and *mg224-R(cherry)* oligonucleotides. Then, the downstream region (which contained an intergenic region between *ftsZ* and MG_225 and a part of MG_225) was also amplified using the *mg224-Dw-F* and *mg224-Dw-R(Cm)* primers. The *mg224-R(cherry)* and *mg224-Dw-F* contained an overhang end that overlapped with the 5' of the *mcherry* reporter and the 3' of the cat resistance marker, respectively. The *mcherry* and *cat* cassette was amplified from the pMG_428:Ch plasmid[3] with the Cherry-F and Cm-R oligonucleotides. Then, the three fragments were joined by SOE-PCR and cloned into an *Eco*RV-digested pBE and later transformed into the G37, *mraZ* and *mraW* strains.

p217YFP. This construction was created to fuse the eYFP fluorescent marker to the MG_217 gene by HR. The upstream region was amplified using the *mg217-YFP-F* and *mg217-YFP-R* oligonucleotides, and the downstream region was also amplified by PCR with the *mg217Dw-PAC-F* and *mg217Dw-R* primers. In this case, the cassette containing the eYFP and *pac* markers was amplified from the pTnMG_428:YFP plasmid[5] using the *YFP-F* and *Pac-R (BamHI)* oligonucleotides. The three fragments were then fused by SOE-PCR, as the *mg217-YFP-R* and *mg217Dw-PAC-F* oligonucleotides contained an overhang that overlapped with the 5' of the *eYFP* marker and the 3' of the puromycin resistance, respectively. Then, this construction was cloned into an *Eco*RV-digested pBE and later transformed into the *mraZ* strain.

pMTnCatmraZ. This plasmid contains a minitransposon carrying a wild-type copy of the *mraZ* allele under the control of its own promoter. It was used to restore the phenotype of the *mraZ* mutant. The *mraZ* ORF was amplified with *MG221P221-F(ApaI)* and *COMmg221-R(XhoI)* primers from gDNA of *M. genitalium*. Then, the fragment was digested with *Xho*I and *Apa*I and cloned into an equally digested *pMTnCat* plasmid[6]. Finally, the construction was transformed into the *mraZ* strain.

pMTnCatmraZW. This plasmid contains a minitransposon carrying wild-type copies of *mraZ* and *mraW* under the control of its own promoter. The two alleles were amplified from gDNA of *M. genitalium* with *MG221P221-F(ApaI)* and *COMmg222-R(XhoI)* primers. The fragment was later digested with *Xho*I and *Apa*I and ligated into a digested *pMTnCat* plasmid. This plasmid was transformed into the *mraZ* strain.

pmraZCOM. This plasmid was used to restore the *mraZ* phenotype by placing a *mraZ* copy after the *ftsZ* gene by HR. The upstream region (*ftsZ*) was amplified using the *mg224-F(cherry)* and the *224mraZ-R* primers. This last oligonucleotide overlapped with the 3' of *ftsZ* and it left a hanging 3' end which was complementary to the 5' of *mraZ*. Then, we amplified the *mraZ* + *Cm*R construction from *pMTnCatmraZ*. This amplification was done using the *mraZ-F* and *Cm-R* primers. The last part of the construct contained the downstream region of *ftsZ* (a short intergenic region of 45 bp and a large part of MG_225) and it was amplified by PCR using the *mg224-Dw-F(Cm)* and *mg224-Dw-R(Cm)* oligonucleotides. The *mg224-Dw-F(Cm)* oligonucleotide had a 5' hanging end which

overlapped with the 3' end of *Cm*R. All three parts were fused together using SOE-PCR and cloned into an *Eco*RV-digested pBE and later transformed into the *mraZ* mutant.

Mutant screening

All the defective strains in this study were screened using Next Generation Sequencing (NGS). We extracted the genomic DNA of each strain and then we obtained libraries for each sample using MiSeq 2x150 flowcells. Next, we performed alignments for each library against their respective reference genome. The alignments showed the absence of large genome rearrangements different to those intended for each mutant (data not shown). We also investigated the presence of small variants like SNPs or INDELS in the alignments. All the strains showed the presence in variable frequencies of a few variants, many of them also found in the G37 strain. However, several variants were not identified in the G37 strain. Most of these new variants were mainly located in MgPar regions. These MgPar regions are repeated regions scattered in the genome that are involved in the generation of antigenic diversity by recombining among them and also with the homologous sequences in the MgPa operon, coding for the main adhesins of *M. genitalium* (Table S3). We consider that the presence of SNPs and INDELs in these regions is a mere consequence of the different passages between the parental G37 strains and the obtained mutant strains. The variable frequencies of these variants might be also a consequence that all mutant strains were derived by colony picking after the transformation experiments.

Table S1. Strains used in this study.

**Strain name**	**Genotype**	**Reference**
G37	Wild-type	ATCC 33530
mraREF	*tetM*	This work
mraZ	ΔMG_221::*tetM*	This work
mraW	ΔMG_222::*tetM*	This work
MG_223	ΔMG_223::*cat*	This work
ftsZ	ΔMG_224::*cat*	This work
dcw	ΔMG_221*::cat, ΔMG_222::cat,* ΔMG_223*::cat, ΔMG_224::cat.*	This work
mraZCOM	ΔMG_221*::tetM, cat.*	This work
mg191	ΔMG_191*::tetM*	Burgos *et al.*, 2006[7]
G37 *ftsZCh*	*cat, ftsZ:mcherry*	This work
mraZftsZCh	ΔMG_221::*tetM, cat, ftsZ:mcherry*	This work
mraWftsZCh	ΔMG_222::*tetM, cat, ftsZ:mcherry*	This work
mg191 ftsZCh	ΔMG_191::*tetM, cat, ftsZ:mcherry*	This work
mraZ ftsZCh 217YFP	ΔMG_221::*tetM, cat, ftsZ:mcherry, pac,* MG_217*:eyfp*	This work
mraZ TnMraZ	ΔMG_221::*tetM*, Tn*Cm*MG_221	This work
mraZ TnMraZW	ΔMG_221::*tetM*, Tn*Cm*MG_221:MG_222	This work

Table S2. Primers used in this study.

	Primer name	Sequence (5'-3')
Mutants	mraZ Upstream F	GTTACACCTACTAACAACAC
	mraZ Upstream R	TTTATTAATTCTAAATACTACAATTCTACAACTTAAATTAACCCTTG
	mraW Downstream F	CGATGAGTGGCAGGGCGGGGCGTAAATGTACAAACCAAAAAATATTAAC
	mraW Downstream R	TTGATAAGTGCAACATTAGC
	TetTer-3'	CAAGGGTTAATTTAAGTTGTAGCTCGAGCTAAAAATCTGTTTTTTGGT
	TetTer-5'	CTTTTTGTCCAAAATGAAATGAATTCTAGTATTTAGAATTAATAAAG
	mraZ-F	ATGCTGCTAGGTACCTTTAATC
	mraZ Up-R	CTACAACTTAAATTAACCCTTG
	mraZProm(Dw-F)	TCATTTCATTTTGGACAAAAAG
	mraW-F	TCATTTCATTTTGGACAAAAAGAAATTTTTATGCTAAGATAAAAGTGTTTAAAAGTGTCGCAAAGTGTGACAAAGTGGAAAAAATGCTAAATAACCAACAGATC
	mraZ-R	ATATTTTTTGGTTTGTACATTTATTTAGCATCTTTCATCC
	mraW(Dw-F)	ATGTACAAACCAAAAAATATTAAC
	mraW+Prom(Dw-F)	TCATTTCATTTTGGACAAAAAGAAATTTTTATGCTAAGATAAAAGTGTTTAAAAGTGTCGCAAAGTGTGACAAAGTGGAAAAAATGTACAAACCAAAAAATATTAAC
	Tc-F (or Cm-F or Pac-F)	CTCGAGTAGTATTTAGAATTAATAAAG (sequence of the MG_438 promoter)
	Cm-R	TTACGCCCCGCCCTGCCAC
	223p438(XhoI)-R	AATTCTAAATACTACTCGAGAGTTATTTAACCAAGCGTTGG
	mg224-F(cherry)	CACTATCCTAATTTAGCAAGTG
	mg224-DW-F(Cm)	GTGGCAGGGCGGGGCGTAATTAATTTAATTTATCGTTTAGAATTGC
	mg224-DW-R(Cm)	CTTTCTGGAGTTGGCAATAATAG
	224mraZ-R	GATTAAAGGTACCTAGCAGCATATTAGTAGATTTGGTTTTGGTGC
	mg217-YFP-F	CAGCAATTTAATCAACCAGG
	mg217-YFP-R	CAGCTCCTCGCCCTTGCTCACGTTATTGTTATTGTTATTGTTATTTTCATAGAAGTCATCACGGTAA
	mg217Dw-PAC-F	CTAGAAAACCTGGTGCTTAAAAAGCGTGTTTTAACTAATGAAA
	mg217Dw-R	TAAGTTGTTTAGCTACATCATC
	Pac-R (BamHI)	GCGGGATCCTTAAGCACCAGGTTTTCTAG
	YFP-F	ATGGTGAGCAAGGGCGAGGA
	MG223 (Up-F)	GTAGCTGAAAGGATGAAAGATG
	MG223 (Up-R)	GTTCAATAAAATAACTTAGGATCCTAGTTTTTTTGGATAACAAAGAG

	MG223 (Dw-F)	TTTATTAATTCTAAATACTACTCGAGTAACTATGGATGAAAATGAAAC
	MG223 (Dw-R)	ATATTAGGGATGGTTGTCACAAAATC
	MG221P221-F(ApaI)	ATTGGGCCCCAAGGGTTAATTTAAGTTGTAGTC
	COMmg221-R(XhoI)	ATTCTCGAGTTATTTAGCATCTTTCATCCTTTC
	COMmg222-R(XhoI)	ATTCTCGAGCTAGTTTTTTTGGATAACAAAGAGC
Screening	mg220(Up-F)	GTGATCCTGATCCAATCCAA
	mg226(Up-R)	ATTAATTCTAAATACTATCTAGAGCCCAACATCAAACATGGTC
qRT-PCR	RTPCRmg177-F	TGAGTGTCCAGCTGGTTTTG
	RTPCRmg177-R	AACCGGGGAAAAGTTAGCAT
	RTPCRmg418-F	TGTTGACGCTAGTGGTTTGG
	RTPCRmg418-R	TTCCACCCATGTATTGAGAGTG
	RTPCRmg430-F	GGAAGCAGTTGGATTGCCTA
	RTPCRmg430-R	ATGCACTCCTCCATTGGAAA
	RTPCRmg221-F	CCTTGATAACAAGAACAGAA
	RTPCRmg221-R	GGAAGTTATTAAAGGTTTGAAA
	RTPCRmg222-F	AGGGTTTGCAGGACACAGTC
	RTPCRmg222-R	TCCCATCAAACTTGGTTATTGA
	RTPCRmg223-F	TGATGATCAAAACCAGTTCAACA
	RTPCRmg223-R	TCAGTTCAGCGAGAACAACAA
	RTPCRmg224-F	GGATGAAAATGAAACTCAATTC
	RTPCRmg224-R	CTTGCTAAATTAGGATAGTGATAA
Sequencing	Fup-24	CGCCAGGGTTTTCCCAGTCACGAC
	Rup-24	TCACACAGGAAACAGCTATGACCA
	TetUp	TTCCTGCATCAACATGAG
	TetDown	GTCGTCCAAATAGTCGGA
	CmUp	CAACGGTGGTATATCCAG
	CmDown	CAGTACTGCGATGAGTGGCA
	PacUp	GTAGCTAATCTAACAGTAGG
	PacDown	GTCCTAGAACTTGGTGTATG

Table S3. Variants (SNPs and INDELS) detected in the genome of analyzed strains. Illumina reads were aligned to the *M. genitalium* G37 reference genome and variants were detected with the VarScan application (Kobolt *et al.*, 2009)[8]. Only variants passing the strand filter, with a P value <0.001, with more than 20 supporting reads and with a frequency higher than 20% were reported by VarScan. NF: Not found; IGR: Intergenic non-coding region. MgPar refers to the different genome regions involved in the generation of antigenic diversity by recombining with the homologous sequences in the MgPa operon (MgPaOp), which codes for the main adhesins of *M. genitalium*.

					Frequency						
Position	Reference Sequence	Alternative Sequence	Type	Locus	G37	*mraZ*	*mraW*	MG_223	*ftsZ*	*dcw*	*mraZCOM*
22285	G	A	SNP	MG_018	27.38	NF	62.16	NF	99.71	100	27.38
36790	T	-AA	INDEL	IGR	38.92	NF	NF	62.16	63.64	58.00	60.80
137879	A	T	SNP	MG_110	20.12	NF	NF	NF	NF	NF	NF
137883	G	T	SNP	MG_110	27.11	NF	NF	NF	NF	NF	NF
167981	C	T	SNP	MgPar2	NF	NF	NF	NF	NF	NF	20.25
168014	A	G	SNP	MgPar2	NF	NF	NF	NF	NF	NF	65.62
168017	A	C	SNP	MgPar2	NF	NF	NF	NF	NF	NF	63.41
168030	T	C	SNP	MgPar2	NF	NF	NF	NF	NF	NF	46.15
168032	A	C	SNP	MgPar2	NF	NF	NF	NF	NF	NF	44.77
168036	G	A	SNP	MgPar2	NF	NF	NF	NF	NF	NF	32.68
168037	C	G	SNP	MgPar2	NF	NF	NF	NF	NF	NF	31.71
168041	G	A	SNP	MgPar2	NF	NF	NF	NF	NF	NF	29.30
168042	T	C	SNP	MgPar2	NF	NF	NF	NF	NF	NF	27.44
169475	A	+TAGTAG	INDEL	MgPar2	21.15	NF	NF	NF	NF	NF	NF
185135	C	A	SNP	MG_146	75.16	100	99.63	99.63	100	100	100
222176	G	C	SNP	MgPaOp	NF	NF	NF	NF	NF	99.73	NF

222502	A	G	SNP	MgPaOp	NF	86.34	NF	86.94	NF	NF	85.87
222505	G	T	SNP	MgPaOp	NF	84.73	NF	86.09	NF	NF	84.94
222507	C	-A	INDEL	MgPaOp	NF	82.88	NF	81.69	NF	NF	80.27
222508	A	G	SNP	MgPaOp	NF	20.78	NF	21.60	NF	NF	23.33
222511	G	+T	INDEL	MgPaOp	NF	70.38	NF	62.38	NF	NF	62.48
222512	A	G	SNP	MgPaOp	NF	63.24	NF	55.83	NF	NF	58.56
222513	A	G	SNP	MgPaOp	NF	81.87	NF	82.08	NF	NF	80.09
224287	G	A	SNP	MgPaOp	NF	NF	NF	NF	NF	NF	30.73
224290	C	A	SNP	MgPaOp	NF	NF	NF	NF	NF	NF	30.35
224303	C	T	SNP	MgPaOp	NF	NF	NF	NF	NF	NF	26.32
224305	C	A	SNP	MgPaOp	NF	NF	NF	NF	NF	NF	26.68
224309	A	G	SNP	MgPaOp	NF	NF	NF	NF	NF	NF	21.43
224310	G	C	SNP	MgPaOp	NF	NF	NF	NF	NF	NF	20.96
224311	C	A	SNP	MgPaOp	NF	NF	NF	NF	NF	NF	20.64
224312	T	C	SNP	MgPaOp	NF	NF	NF	NF	NF	NF	21.63
224314	A	G	SNP	MgPaOp	NF	NF	NF	NF	NF	NF	21.46
224315	C	T	SNP	MgPaOp	NF	NF	NF	NF	NF	NF	22.12
224532	A	+TAG	INDEL	MgPaOp	NF	NF	NF	NF	NF	65.64	NF
227128	A	-TAGTAG	INDEL	MgPaOp	36.10	NF	NF	NF	24.67	22.84	NF
349545	C	T	SNP	MgPar8	NF	23.95	36.95	36.95	NF	NF	30.52
349557	G	A	SNP	MgPar8	NF	25.69	37.69	37.69	NF	NF	32.69
349560	A	G	SNP	MgPar8	NF	26.17	37.44	37.44	NF	NF	32.60
349590	G	A	SNP	MgPar8	NF	68.97	62.62	62.62	NF	NF	66.38
349593	T	G	SNP	MgPar8	NF	68.77	63.45	63.45	NF	NF	65.92
349595	C	+A	INDEL	MgPar8	NF	65.33	55.17	55.17	NF	NF	58.42
349598	G	-T	INDEL	MgPar8	NF	62.96	53.94	53.94	NF	NF	55.43
349600	G	A	SNP	MgPar8	NF	65.04	58.78	58.78	NF	NF	58.64
349601	G	A	SNP	MgPar8	NF	62.20	58.13	58.13	NF	NF	57.31

429304	A	-TAGTAG	INDEL	MgPar9	NF	40.00	34.55	34.55	NF	NF	36.25
429966	C	-CTTCTTCTTCTTCTTCTT	INDEL	MgPar9	48.08	NF	21.36	21.36	NF	NF	NF
429993	T	-CTTCTTCTTCTTCTTCTTCTTCTA	INDEL	MgPar9	NF	NF	NF	NF	NF	22.13	NF
429996	T	-CTTCTTCTTCTTCTTCTTCTA	INDEL	MgPar9	48.08	NF	NF	NF	23.36	69.40	NF
429999	T	A	SNP	MgPar9	53.60	57.75	NF	51.73	57.07	73.49	51.20
430002	T	A	SNP	MgPar9	58.55	56.16	50.82	50.82	60.77	72.09	52.14
430005	T	A	SNP	MgPar9	56.28	53.61	49.17	49.17	57.21	67.94	48.74
430008	T	A	SNP	MgPar9	50.46	47.72	45.04	45.04	50.76	58.63	NF
430011	T	A	SNP	MgPar9	46.57	43.81	40.12	40.12	43.47	52.14	NF
430014	T	A	SNP	MgPar9	40.56	39.78	35.60	35.60	37.24	42.28	36.30
430017	A	T	SNP	MgPar9	55.62	NF	NF	NF	66.96	89.70	NF
432007	A	C	SNP	MG_340	99.81	100	100	100	99.88	100	99.87
447366	A	G	SNP	MG_349	75.77	100	99.15	99.15	99.57	100	100
580070	A	+AAATACT	INDEL	IGR	NF	NF	NF	NF	25.00	NF	NF
580073	A	T	SNP	IGR	33.33	NF	NF	NF	32.14	50.00	NF
580075	A	G	SNP	IGR	NF	NF	NF	NF	45.00	NF	NF
580076	C	T	SNP	IGR	42.86	NF	NF	NF	NF	NF	NF

Table S4. Differentially expressed proteins in a *mraZ* background. The Area Under the Curve (AUC) for the three best peptides of the proteins in the three strains is specified as well as the Fold Change (FC) with respect to the G37 strain. The gene name is stated when characterized. Only proteins with a biologically significant fold change (>2, <0.5) compared to the G37 strain were considered and these values are highlighted in bold. ND stands for Not Detected.

Overexpressed							
Locus tag	Gene	Gene product	G37	*mraZ*		*mraW*	
			AUC	AUC	FC	AUC	FC
MG_224	*ftsZ*	Cell division protein *ftsZ*	$4.40 \cdot 10^6$	$1.10 \cdot 10^8$	**24.96**	$8.50 \cdot 10^6$	1.93
MG_222	*mraW*	Ribosomal RNA small subunit methyltransferase H	$4.17 \cdot 10^7$	$5.95 \cdot 10^8$	**14.28**	ND	–
MG_091	*ssb*	Single-stranded DNA-binding protein	$1.10 \cdot 10^7$	$1.53 \cdot 10^8$	**13.94**	$1.38 \cdot 10^8$	**12.58**
MG_516		UPF0154 protein	$7.70 \cdot 10^6$	$5.65 \cdot 10^7$	**7.34**	$4.90 \cdot 10^6$	0.64
MG_306		Uncharacterized membrane protein	$3.10 \cdot 10^6$	$1.65 \cdot 10^7$	**5.32**	$6.80 \cdot 10^6$	**2.19**
MG_318	*p32*	P32 adhesin	$1.54 \cdot 10^8$	$6.57 \cdot 10^8$	**4.28**	$1.51 \cdot 10^8$	0.99
MG_042	*potA*	Spermidine/putrescine import ATP-binding protein	$3.65 \cdot 10^7$	$1.23 \cdot 10^8$	**3.38**	$9.18 \cdot 10^7$	**2.52**
MG_233		Uncharacterized protein	$8.40 \cdot 10^6$	$2.60 \cdot 10^7$	**3.10**	$3.85 \cdot 10^7$	**4.58**
MG_326		DegV domain-containing protein	$5.22 \cdot 10^7$	$1.04 \cdot 10^8$	**2.00**	$4.82 \cdot 10^7$	0.92

Underexpressed							
Locus tag	Gene	Gene product	G37	*mraZ*		*mraW*	
			AUC	AUC	FC	AUC	FC
MG_261	*dnaE*	DNA polymerase III subunit alpha	$3.48 \cdot 10^7$	$1.69 \cdot 10^7$	**0.48**	$2.27 \cdot 10^7$	0.65
MG_445	*trmD*	tRNA (guanine-N(1)-)-methyltransferase	$4.66 \cdot 10^7$	$2.20 \cdot 10^7$	**0.47**	$3.28 \cdot 10^7$	0.70
MG_075		Uncharacterized protein	$1.92 \cdot 10^8$	$8.83 \cdot 10^7$	**0.46**	$1.10 \cdot 10^8$	0.57
MG_460	*ldh*	L-lactate dehydrogenase	$5.57 \cdot 10^9$	$2.47 \cdot 10^9$	**0.44**	$2.55 \cdot 10^9$	**0.46**
MG_077	*oppB*	Oligopeptide transport system permease protein	$9.22 \cdot 10^7$	$3.88 \cdot 10^7$	**0.42**	$4.50 \cdot 10^7$	**0.49**
MG_320		Uncharacterized membrane protein	$3.85 \cdot 10^7$	$1.36 \cdot 10^7$	**0.35**	$3.55 \cdot 10^7$	0.92
MG_068		Uncharacterized lipoprotein	$1.41 \cdot 10^7$	$2.70 \cdot 10^6$	**0.19**	ND	-
MG_289	*p37*	High affinity transport system protein p37	$1.14 \cdot 10^8$	$1.83 \cdot 10^7$	**0.16**	$8.17 \cdot 10^7$	0.72

Table S5. Proteins detected in the *mraZ* mutant and not in the wild-type strain and vice versa.

Detected in *mraZ* and not in G37					
Locus tag	Gene	Gene product	G37	*mraZ*	*mraW*
			AUC	AUC	AUC
MG_027	*nusB*	Transcription termination/antitermination protein	ND	$9.20\cdot10^{7}$	$6.70\cdot10^{7}$
MG_452		Uncharacterized membrane protein	ND	$4.03\cdot10^{7}$	ND
MG_044	*potC*	Spermidine/putrescine transport system permease protein	ND	$1.70\cdot10^{7}$	ND
MG_057	*rnmV*	Ribonuclease M5	ND	$1.40\cdot10^{7}$	ND
MG_011		Uncharacterized protein	ND	$1.28\cdot10^{7}$	ND
MG_447		Uncharacterized membrane protein	ND	$1.20\cdot10^{7}$	$9.30\cdot10^{6}$
MG_223		Uncharacterized protein	ND	$7.88\cdot10^{6}$	ND
MG_463	*rsmA*	Ribosomal RNA small subunit methyltransferase A	ND	$6.90\cdot10^{6}$	ND
MG_360		DNA polymerase involved in DNA repair	ND	$6.85\cdot10^{6}$	$7.00\cdot10^{6}$
MG_477		Uncharacterized protein	ND	$6.60\cdot10^{6}$	ND
MG_411		Phosphate transport system permease protein PstA homolog	ND	$6.30\cdot10^{6}$	ND
MG_043	*potB*	Spermidine/putrescine transport system permease protein	ND	$3.15\cdot10^{6}$	$1.30\cdot10^{6}$

Detected in G37 and not in *mraZ*					
Locus tag	Gene	Gene product	G37	*mraZ*	*mraW*
			AUC	AUC	AUC
MG_221	*mraZ*	Transcriptional regulator MraZ	$4.12\cdot10^{8}$	ND	$4.65\cdot10^{8}$
MG_226		Amino acid-polyamine-organocation (APC) permease family protein	$1.80\cdot10^{7}$	ND	ND
MG_147		Uncharacterized membrane protein	$7.80\cdot10^{6}$	ND	$8.40\cdot10^{6}$
MG_440		Uncharacterized lipoprotein	$4.40\cdot10^{6}$	ND	ND

Table S6. Differentially expressed proteins in the *mraW* mutant. Only proteins with a biologically significant fold change (>2, <0.5) compared to the G37 strain were considered and these values are highlighted in bold. AUC stands for Area Under the Curve.

Overexpressed							
Locus tag	Gene	Gene product	G37	*mraZ*		*mraW*	
			AUC	AUC	FC	AUC	FC
MG_091	*ssb*	Single stranded DNA binding protein	$1.10\cdot10^{7}$	$1.53\cdot10^{8}$	**13.94**	$1.38\cdot10^{8}$	**12.58**
MG_233		Uncharacterized protein	$8.40\cdot10^{6}$	$2.60\cdot10^{7}$	**3.10**	$3.85\cdot10^{7}$	**4.58**
MG_042	*potA*	Spermidine/putrescine import ATP-binding protein	$3.65\cdot10^{7}$	$1.23\cdot10^{8}$	**3.38**	$9.18\cdot10^{7}$	**2.52**
MG_306		Uncharacterized membrane protein	$3.10\cdot10^{6}$	$1.65\cdot10^{7}$	**5.32**	$6.80\cdot10^{6}$	**2.19**
MG_473	*rpmG2*	50S ribosomal protein L33 type 2	$2.70\cdot10^{7}$	$4.85\cdot10^{7}$	1.80	$5.80\cdot10^{7}$	**2.15**

Underexpressed							
Locus tag	Gene	Gene product	G37	*mraZ*		*mraW*	
			AUC	AUC	FC	AUC	FC
MG_179	*ecfA1*	Energy-coupling factor transporter ATP-binding protein EcfA1	$1.24\cdot10^{8}$	$9.70\cdot10^{7}$	0.78	$6.13\cdot10^{7}$	**0.50**
MG_077	*oppB*	Oligopeptide transport system permease protein	$9.22\cdot10^{7}$	$3.88\cdot10^{7}$	**0.42**	$4.50\cdot10^{7}$	**0.49**
MG_332		Uncharacterized protein	$1.92\cdot10^{8}$	$1.60\cdot10^{8}$	0.83	$9.37\cdot10^{7}$	**0.49**
MG_250	*dnaG*	DNA primase	$3.08\cdot10^{7}$	$2.47\cdot10^{7}$	0.80	$1.49\cdot10^{7}$	**0.48**
MG_373		Uncharacterized protein	$6.00\cdot10^{7}$	$6.10\cdot10^{7}$	1.02	$2.87\cdot10^{7}$	**0.48**
MG_206	*uvrC*	UvrABC system protein C	$4.53\cdot10^{7}$	$3.67\cdot10^{7}$	0.81	$2.14\cdot10^{7}$	**0.47**
MG_366		Uncharacterized protein	$1.90\cdot10^{7}$	$1.63\cdot10^{7}$	0.86	$8.93\cdot10^{6}$	**0.47**
MG_197	*rpmI*	50S ribosomal protein L35	$2.80\cdot10^{8}$	$2.30\cdot10^{8}$	0.82	$1.31\cdot10^{8}$	**0.47**
MG_304		Putative ABC transporter ATP-binding protein	$2.27\cdot10^{7}$	$1.38\cdot10^{7}$	0.61	$1.04\cdot10^{7}$	**0.46**
MG_460	*ldh*	L-lactate dehydrogenase	$5.57\cdot10^{9}$	$2.47\cdot10^{9}$	**0.44**	$2.55\cdot10^{9}$	**0.46**
MG_388	*ybeY*	Endoribonuclease YbeY	$1.30\cdot10^{7}$	$1.10\cdot10^{7}$	0.85	$5.80\cdot10^{6}$	**0.45**
MG_047	*metK*	S-adenosylmethionine synthase, AdoMet synthase	$6.75\cdot10^{7}$	$4.02\cdot10^{7}$	0.60	$3.00\cdot10^{7}$	**0.44**
MG_252		Uncharacterized tRNA/rRNA methyltransferase	$5.18\cdot10^{7}$	$2.98\cdot10^{7}$	0.58	$2.27\cdot10^{7}$	**0.44**
MG_040		Uncharacterized lipoprotein	$5.07\cdot10^{8}$	$3.27\cdot10^{8}$	0.64	$2.13\cdot10^{8}$	**0.42**
MG_103		Probable transcriptional regulator WhiA	$1.66\cdot10^{7}$	$1.16\cdot10^{7}$	0.70	$6.90\cdot10^{6}$	**0.42**
MG_024	*ychF*	Ribosome-binding ATPase	$8.12\cdot10^{7}$	$4.13\cdot10^{7}$	0.51	$3.33\cdot10^{7}$	**0.41**

Table S7. Subset of proteins above the detection threshold in *mraW* and not detected in G37 and vice versa.

Detected in *mraW* and not in G37					
Locus tag	Gene	Gene product	G37	*mraZ*	*mraW*
			AUC	AUC	AUC
MG_027	*nusB*	Transcription termination/antitermination protein	ND	$9.20 \cdot 10^{7}$	$6.70 \cdot 10^{7}$
MG_447		Uncharacterized membrane protein	ND	$1.20 \cdot 10^{7}$	$9.30 \cdot 10^{6}$
MG_521		Uncharacterized membrane protein	ND	ND	$9.30 \cdot 10^{6}$
MG_360		DNA polymerase involved in DNA repair	ND	$6.85 \cdot 10^{6}$	$7.00 \cdot 10^{6}$
MG_358	*ruvA*	Holliday junction ATP-dependent DNA helicase RuvA	ND	ND	$6.90 \cdot 10^{6}$
MG_291	*p69*	ABC transport system permease protein p69	ND	ND	$4.80 \cdot 10^{6}$
MG_339	*recA*	Protein RecA	ND	ND	$3.70 \cdot 10^{6}$
MG_043	*potB*	Spermidine/putrescine transport system permease protein	ND	$3.15 \cdot 10^{6}$	$1.30 \cdot 10^{6}$

Detected in G37 and not in *mraW*					
Locus tag	Gene	Gene product	G37	*mraZ*	*mraW*
			AUC	AUC	AUC
MG_222	*mraW*	Ribosomal RNA small subunit methyltransferase H	$4.17 \cdot 10^{7}$	$5.95 \cdot 10^{8}$	ND
MG_184		Uncharacterized adenine-specific methylase	$2.40 \cdot 10^{7}$	$1.50 \cdot 10^{7}$	ND
MG_226		Amino acid-polyamine-organocation (APC) permease family protein	$1.80 \cdot 10^{7}$	ND	ND
MG_068		Uncharacterized lipoprotein	$1.41 \cdot 10^{7}$	$2.70 \cdot 10^{6}$	ND
MG_294		Uncharacterized protein	$1.27 \cdot 10^{7}$	$1.50 \cdot 10^{7}$	ND
MG_293		Glycerophosphoryl diester phosphodiesterase family protein	$1.18 \cdot 10^{7}$	$6.85 \cdot 10^{6}$	ND
MG_505		Putative pre-16S rRNA nuclease	$1.02 \cdot 10^{7}$	$1.30 \cdot 10^{7}$	ND
MG_440		Uncharacterized lipoprotein	$4.40 \cdot 10^{6}$	ND	ND

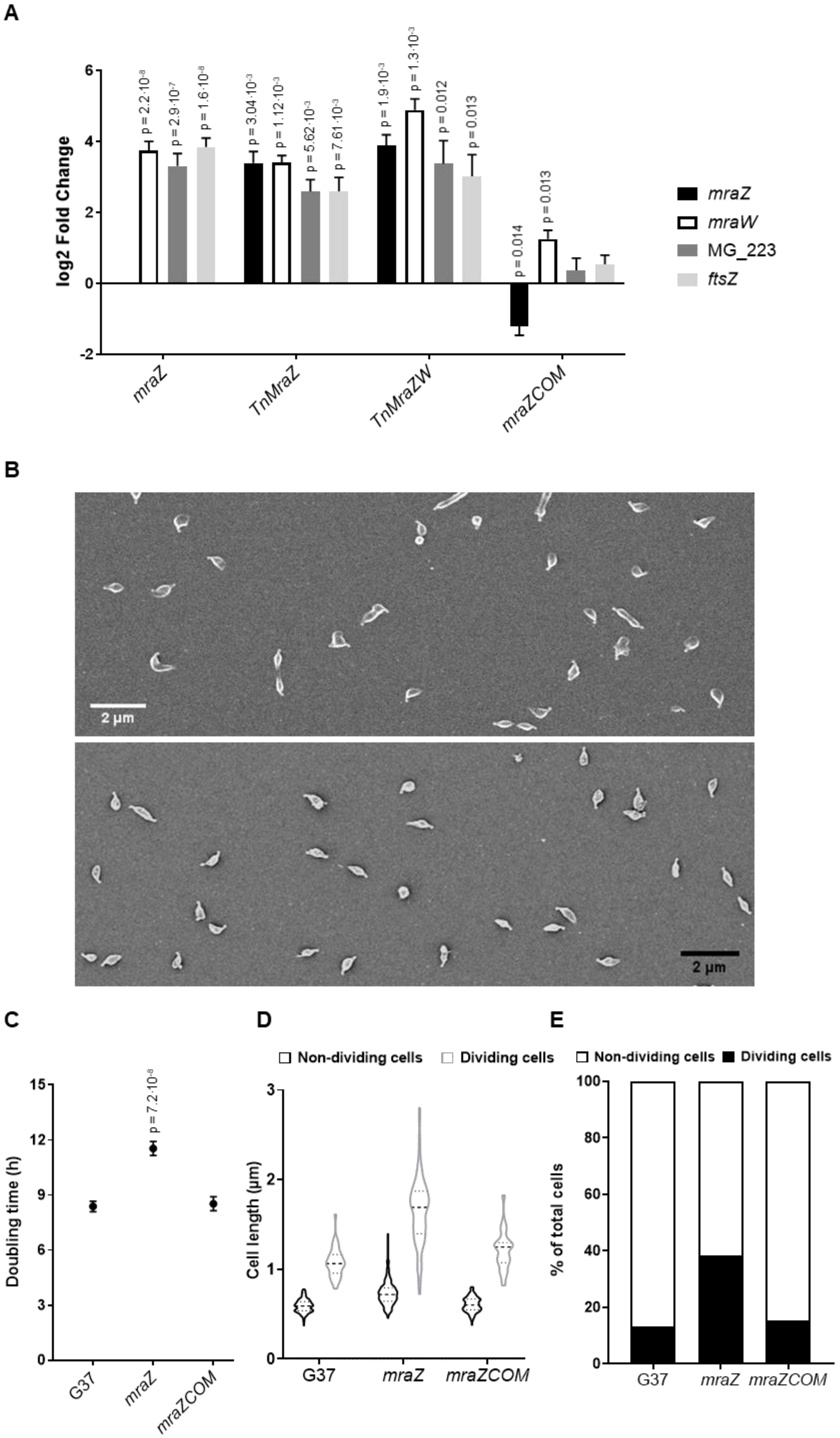
A
log2 Fold Change
6
4
2
0
-2
p = 2.2·10-8
p = 2.9·10-7
p = 1.6·10-8
p = 3.04·10-3
p = 1.12·10-3
p = 5.62·10-3
p = 7.61·10-3
p = 1.9·10-3
p = 1.3·10-3
p = 0.012
p = 0.013
p = 0.014
p = 0.013
mraZ
TnMraZ
TnMraZW
mraZCOM
mraZ
mraW
MG_223
ftsZ
B
2 µm
2 µm
C
Doubling time (h)
15
12
9
6
3
0
p = 7.2·10-8
G37
mraZ
mraZCOM
D
Non-dividing cells
Dividing cells
Cell length (µm)
3
2
1
0
G37
mraZ
mraZCOM
E
Non-dividing cells
Dividing cells
% of total cells
100
80
60
40
20
0
G37
mraZ
mraZCOM

Supplemental Figure S1. Restoration of the wild-type phenotype after re-introducing a *mraZ* ectopic copy. **(A)** qRT-PCR data of the *mraZ* mutant, the *mraZ* mutant complemented with an ectopic copy of *mraZ* in a minitransposon (*TnMraZ*), the mutant complemented with ectopic copies of *mraZ* and *mraW* in a minitransposon (*TnMraZW*) and the strain carrying the new copy of *mraZ* after the *ftsZ* gene (*mraZCOM*). Bars represent the mean of at least three independent biological repeats. Statistically significant values assessed by a paired T-test are above the corresponding bar if biologically significant (log2 fold change ± 1). **(B)** SEM micrographs of the wild-type strain (top panel) and the *mraZCOM* complemented strain (bottom panel). Scale bars represent 2 µm. **(C)** Doubling time of the *mraZCOM* strain compared to its parental (*mraZ*) and wild-type strains. Statistical significance was assessed using a paired T-test. Significant values ($p < 0.05$) are indicated above its corresponding experimentally determined value. **(D)** Length of non-dividing or dividing cells in the G37, *mraZ* and *mraZCOM* strains. Several SEM micrographs of each strain were analyzed using the ImageJ software. An average of 280 cells were measured for each strain. **(E)** Frequence of non-dividing and dividing cells as observed in SEM micrographs for the three tested strains. An average of 900 cells were analyzed for each strain.

References

1. Martínez-Torró, C., Torres-Puig, S., Monge, M., Sánchez-Alba, L., González-Martín, M., Marcos-Silva, M., Perálvarez-Marín, A., Canals, F., Querol, E., Piñol, J., & Pich, O. Q.. Transcriptional response to metal starvation in the emerging pathogen *Mycoplasma genitalium* is mediated by Fur-dependent and –independent regulatory pathways. *Emerging Microbes and Infections*, 9(1), 5–19 (2020).

2. Pich, O. Q., Burgos, R., Planell, R., Querol, E. & Piñol, J. Comparative analysis of antibiotic resistance gene markers in *Mycoplasma genitalium*: application to studies of the minimal gene complement. *Microbiology* 152, 519–527 (2006).

3. Kingsford, C. L., Ayanbule, K. & Salzberg, S. L. Rapid, accurate, computational discovery of Rho-independent transcription terminators illuminates their relationship to DNA uptake. *Genome Biol.* 8, R22 (2007).

4. Torres-Puig, S., Broto, A., Querol, E., Piñol, J. & Pich, O. Q. A novel sigma factor reveals a unique regulon controlling cell-specific recombination in *Mycoplasma genitalium*. *Nucleic Acids Res.* 43, 4923–4936 (2015).

5. Torres-Puig, S. *et al.* Activation of sigma20-dependent recombination and horizontal gene transfer in *Mycoplasma genitalium*. *DNA Res.* (2018). doi:10.1093/dnares/dsy011.

6. Calisto, B. M. *et al.* The EAGR box structure: a motif involved in Mycoplasma motility. *Mol. Microbiol.* 86, 382–393 (2012).

7. Burgos, R., Pich, O. Q., Ferrer-Navarro, M., Baseman, J. B., Querol, E., & Piñol, J.. Mycoplasma genitalium P140 and P110 cytadhesins are reciprocally stabilized and required for cell adhesion and terminal-organelle development. *Journal of Bacteriology, 18*8(24), 8627–8637 (2006).

8. Koboldt DC, Chen K, Wylie T, Larson DE, McLellan MD, Mardis ER, Weinstock GM, Wilson RK, & Ding L VarScan: variant detection in massively parallel sequencing of individual and pooled samples. *Bioinformatics*, 25 (17), 2283-5 PMID: 19542151 (2009).
